# Cyanobacterial mats and their associated microbiomes in saline and freshwater lakes from the Bolivian Altiplano

**DOI:** 10.3389/fmicb.2025.1650455

**Published:** 2025-07-23

**Authors:** Guilherme Scotta Hentschke, Miguel Semedo, Jimmy Ciancas, Claudia Hoepfner, Daniel Guzmán, Daniela S. Rivera, Vitor M. Vasconcelos

**Affiliations:** ^1^CIIMAR/CIMAR-LA-Terminal de Cruzeiros do Porto de Leixões, Matosinhos, Portugal; ^2^Fundación PROINPA, Cochabamba, Bolivia; ^3^Faculty of Sciences and Technology, Center of Biotechnology, Universidad Mayor de San Simón, Cochabamba, Bolivia; ^4^GEMA Center for Genomics, Ecology and Environment, Universidad Mayor, Santiago, Chile; ^5^Department of Biology, Faculty of Sciences, University of Porto, Porto, Portugal

**Keywords:** bacteria, biodiversity, microbiome, extremophiles, functional analysis, Cyanobacteria

## Abstract

The Bolivian Altiplano presents extreme environmental conditions, including high altitude, intense UV radiation, low precipitation, freezing temperatures, and saline to alkaline waters. Despite these harsh settings, cyanobacteria thrive in microbial mats, although their diversity remains poorly characterized. This study aimed to explore the morphological and molecular diversity of cyanobacterial mats and their associated microbiomes in saline and freshwater ecosystems of the Bolivian Altiplano. Morphological analyses revealed seven distinct cyanobacterial morphotypes affiliated with Nostocaceae, Coleofasciculaceae, Rivulariaceae, and Microcoleaceae. Amplicon-based analysis of the 16S rRNA gene identified 4.113 ASV for the bacterial community. Of these, 310 were identified as Cyanobacteria, with 134 classified as Cyanophyceae assigned to 32 genera. Phylogenetic reconstruction and sequence identity comparisons resolved 42 cyanobacterial genera across nine orders. Moreover, 30 ASVs grouped into 16 clades unrelated to any known genus, suggesting the presence of potentially novel cyanobacterial lineages. The microbiome associated with these mats was dominated by Alphaproteobacteria, Bacteroidia, Gammaproteobacteria, Clostridia, Cyanophyceae, and Campylobacteria. Functional predictions based on 16S rRNA gene profiles indicated a predominance of phototrophic and chemoheterotrophic metabolisms, along with sulfur respiration, nitrogen fixation, nitrate and nitrite reduction, and fermentation pathways. Notably, nitrogen-fixing cyanobacteria and bacterial groups with bioremediation potential were prevalent, highlighting the ecological importance and possible biotechnological applications of these microbial consortia. This is the first comprehensive metabarcoding analysis of cyanobacterial mats from Bolivia, including their associated microbiomes. Many new bacterial and cyanobacterial taxa remain to be described in these ecosystems. Based on the functional genomic analysis, this work also highlights the great unexplored biotechnological potential of Bolivia’s extreme environments and the functional roles of microbial mats in biogeochemical cycling under polyextreme conditions.

## 1 Introduction

Extreme environments exist as a substantial portion of Earth’s habitable zone. They present a myriad of challenges for life, pushing the boundaries of what may be considered habitable. These challenges include extreme temperatures, pressure, high salinity, and pH, as well as oxygen and nutrient deprivation (Rothschild and Mancinelli, 2001). Despite these conditions, some extremophile organisms are capable of thriving and emerge as examples of adaptation and resilience. Across these environments, we can find all three domains of life, however, bacteria and archaea are the most abundant due to their exceptional diversity and adaptability (Shu and Huang, 2022; Thakur et al., 2022).

Among these extreme environments, the Bolivian Altiplano stands out as a particularly intriguing site. The Altiplano, a closed drained basin, spans 200,000 km^2^ and harbors freshwater and saltwater lakes, as well as extended salt flats (Fornari et al., 2001). The climate in the region is arid, with water evaporation rates largely exceeding annual rainfall. Temperatures can reach as low as −30°C in winter and 20–25°C in summer, whereas the lakes contain low water levels or are desiccated (Risacher and Fritz, 1991). It is also a high-altitude region exposed to elevated UV radiation (Cabrol et al., 2014; Daga-Quisbert et al., 2023). In the southern part of the Bolivian Altiplano, the Lipez region is home to both temporary and permanent saline lakes, including Laguna Colorada, Laguna Verde, and Laguna Hedionda, among others.

The biodiversity of these ecosystems has been poorly described, but early studies noted the presence of algae (Servant-Vidalry, 1978, 1984) and benthic invertebrates (Dejoux, 1993). Over the past 20 years, considerable efforts have been made to characterize and conserve the microbial ecosystems that thrive in these saline lakes (Cabrol et al., 2009; Fleming and Bebout, 2010; Guzmán et al., 2010; Albarracin et al., 2015; Vignale et al., 2021), but their extreme conditions and difficult accessibility, have left them largely understudied.

Over the last decade, the development of culture-independent techniques for studying microbial ecosystems, such as high-throughput sequencing and metagenomics, have represented a milestone in our understanding of such environments. These techniques can improve our knowledge on the evolution of both bacteria (Andrei et al., 2015) and archaea (Bodaker et al., 2010; Narasingarao et al., 2012; Adam et al., 2017), thus contributing to the conservation of these ecosystems.

According to that, our study aimed to delve deeper into the microbial community structure of the saline and freshwater lakes in the southern Bolivian Altiplano, in order to understand the distinct biodiversity present in these two ecosystems. Combining high-throughput metabarcoding techniques with comprehensive morphological analysis we sought to elucidate the morphological and genetic diversity of cyanobacterial mats and their associated microbiomes. Our findings underscore the adaptability of these microorganisms and highlight the strong influence of the environmental conditions on their structure. Moreover, it emphasized the importance of preserving these unique and underexplored ecosystems, advancing our understanding of microbial evolution and resilience.

## 2 Materials and methods

### 2.1 Sampling and characterization of the studied habitats

Sampling was performed between 24th and 29th August 2023 in the framework of an international expedition with teams from Bolivia, Mexico, Portugal, Spain and Slovenia coordinated by the Universidad Mayor de San Simón and Fundación PROINPA, from Cochabamba, Bolivia.

**Figure 1:**
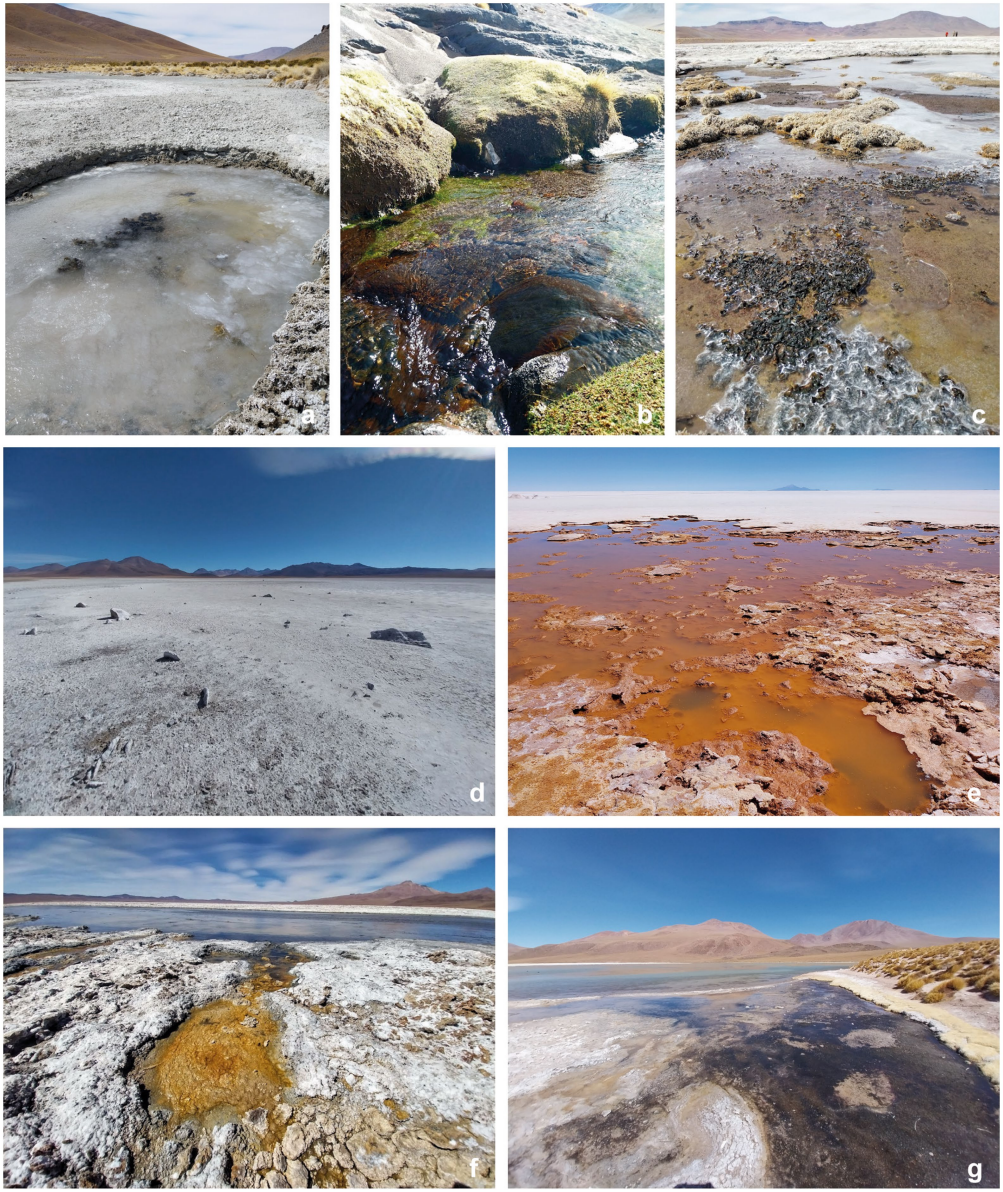
Sampling sites. **(a)** Laguna Chojllas Pequeña; **(b)** Laguna Colorada; **(c)** Laguna Coruto; **(d)** Laguna Mama Khumu; **(e)** Salar de Uyuni, **(f,g)** Laguna Pastos Grandes.

In order to obtain a robust representation of the microbial communities across the lakes, the sampling strategy was designed to mitigate potential biases by collecting several randomly selected samples per lake and combining them into a pooled sample. A total of eight lagoons were sampled, including four freshwater (salinity < 0.5%) and four saline lagoons (salinity > 0.5%) (Table 1; Figure 1). This sampling strategy ensures that each replicate represents an entire lake rather than a single localized point, minimizing the effects of spatial heterogeneity. These sites were characterized by extreme environmental conditions, severe temperature fluctuations, low precipitation, and altitudes ranging from 3,656 to 4,430 m (Cabrol et al., 2009, 2014). They were specifically selected to investigate the impact of salinity on microbial diversity and functional adaptations. This approach provides critical insights into ecosystem resilience and biogeochemical processes across freshwater and saline ecosystem enhancing our understanding of microbial dynamics in extreme environments.

**Table 1:** Sampling sites and Cyanophyceae biodiversity.

Sample location	Description of habitat	GPS coordinates	Morphotypes	Cyanophyceae genera confirmed by phylogeny	Cyanophyceae genera identified by metabarcoding	Associated Microbiome
Salar de Uyuni (GBC221)	Shore of saline flat pH = 6.3	20°18′41.98″S 66°59′50.89″W	*Nostoc* sp1.	*Anagnostidinema, Fulbrightiella, Macrochaete, Nodularia, Mojavia, Nostoc, Unknown genus 6, Purpureonostoc, Pseudoaliinostoc, Cyanocohniella, Anabaena, Trichormus, Dulcicalothrix, Cylindrospermum, Toxifilum, Nodosilinea, Haloleptolyngbya, Cyanobium,* Unknown genus 10	*Geitlerinema* LD9, *Calothrix* PCC-6303, *Nodularia* PCC-9350, *Nostoc* PCC-73102, *Nostoc* PCC-7524, *Nodosilinea* PCC-7104, *Cyanobium* PCC-6307, Sericytochromatia	Alphaproteobacteria, Bacteroidia, Gammaproteobacteria, Clostridia, Bacilli and Campylobacteria in similar proportions.
Salar de Uyuni (GBC222)			*Nostoc* sp2.	*Fulbrightiella, Macrochaete, Microchaete, Mojavia, Nostoc,* Unknown genus 6, *Purpureonostoc, Pseudoaliinostoc, Nodosilinea, Haloleptolyngbya, Cyanobium*	*Calothrix* PCC-6303, *Desmonostoc* PCC-7422, *Nostoc* PCC-73102, *Nostoc* PCC-7524, *Nodosilinea* PCC-7104, *Cyanobium* PCC-6307	Alphaproteobacteria, Bacteroidia, Gammaproteobacteria, Clostridia, Bacilli and Campylobacteria in similar proportions.
Catal Lake (GBC223)	Shore of freshwater stream pH = 7.9	21°35′41.31″S 67°35′48.78″W	*Nostoc* sp2.	Unknown genus 10, Unknown genus 8, *Nodularia, Mojavia, Nostoc,* Unknown genus 6, *Cyanocohniella, Anabaena, Trichormus, Dulcicalothrix, Cylindrospermum, Timaviella, Cyanobium*	Cyanobacterales, *Nodularia* PCC-9350, *Nostoc* PCC-73102, Nostocaceae, *Phormidium* CYN64, *Cyanobium* PCC-6307	Dominated by *Weissella* (Bacilli)
Laguna Colorada (GBC224)	Shore of warm freshwater stream pH = 9.7	22°10′15.45″S 67°48′19.50″W	*Microcoleus* sp.	Unknown genus 1, *Limnoraphis*, *Microcoleus*, *Salileptolyngbya, Phormidesmis,* Unknown genus 9, Unknown genus 16, *Timaviella*	Phormidiaceae, Leptolyngbyaceae, *Phormidium* CYN64	Dominated by Bacteroidia
Laguna Chalviri (GBC225)	Shore of warm freshwater stream pH = 8.5	22°32′8.57″S 67°38′56.32″W	*Nostoc* sp3.	*Anagnostidinema,* Unknown genus 10, Unknown genus 8, *Potamosiphon, Limnococcus,* Unknown genus 7, *Microcystis, Synechocystis, Mojavia, Nostoc,* Unknown genus 6, *Purpureonostoc, Pseudoaliinostoc, Rivularia, Thermoleptolyngbya, Nodosilinea, Haloleptolyngbya,* Unknown genus 5, *Altericista, Cyanobium, Parasynechococcus,* Unknown genus 10	*Geitlerinema* LD9, Cyanobacteriales, *Microseira* Carmichael-Alabama, Chroococcales cyanobacterium—*Gloeocapsa*, *Gleocapsa*, *Microcystis* PCC-7914, Synechocystis PCC-6803, *Nostoc* PCC-73102, *Nostoc* PCC-7524, *Rivularia* PCC-7116, *Geitlerinema* PCC-8501, *Nodosilinea* PCC-7104, *Synechococcus* PCC-7502, SepB-3, *Cyanobium* PCC-6307, *Synechococcus* sp., Sericytochromatia	Dominated by Alphaproteobacteria and “other bacteria”
Laguna Chojllas Pequeña (GBC226)	Shore of frozen freshwater lake pH = 7.7	22°21′22.4″S 67°05′50.1″W	*Nostoc* sp4.	*Mojavia, Nostoc,* Unknown genus 6, *Chamaesiphon*	*Nostoc* PCC-73102, *Calothrix* KVSF5	Dominated by *Pseudomonas* (Gammaproteobacteria)
Laguna Coruto (GBC227)	Shore of saline lake pH = 8.6	22°25′41.7″S 67°00′35.2″W	*Nostoc* sp4.	*Mojavia, Nostoc,* Unknown genus 6, *Nodosilinea, Haloleptolyngbya*	*Nostoc* PCC-73102, *Nodosilinea* PCC-7104	Dominated by *Clostridium* (Clostridia)
Laguna Mama Khumu (GBC228)	Shore of saline lake pH = 9	22°15′59.2″S 67°04′38.1″W	*Coleofasciculus* sp.	*Coleofasciculus, Pycnacronema, Jaaginema*	*Coleofasciculus* PCC-7420, Paraspirulinaceae	Dominated by Alphaproteobacteria, Bacteroidia, Deinococci and “other bacteria”
Laguna Pastos Grandes (GBC229)	Shore of saline lake pH = 7.5	21°35′43.63″S 67°50′30.75″W	*Nostoc* sp1.	*Mojavia, Nostoc,* Unknown genus 6, *Rivularia,* Unknown genus 3	*Nostoc* PCC-73102, *Rivularia* PCC-7116, *Schizothrix* LEGE	Dominated by Alphaproteobacteria, Bacteroidia, Gammaproteobacteria in similar proportions
Laguna Pastos Grandes (GBC230)			Heterocytous fasciculated morphotype	*Crocosphaera,* Unknown genus 14, *Rivularia, Toxifilum, Nodosilinea, Haloleptolyngbya,* Unknown genus 3	*Microcystaceae*, *Rivularia* PCC-7116, *Nodosilinea* PCC-7104, Schizothrix LEGE	Dominated by Alphaproteobacteria, Bacteroidia, Clostridia and “other bacteria”
Laguna Pastos Grandes (GBC231)			Heterocytous fasciculated morphotype	Unknown genus 4, *Rivularia,* Unknown genus 2, *Toxifilum,* Unknown genus 3, Unknown genus 10	*Symphothece* PCC-7002, *Rivularia* PCC-7116, Oscillatoriaceae, *Schizothrix* LEGE, Sericytochromatia	Dominated by Alphaproteobacteria, Bacteroidia, Gammaproteobacteria and “other bacteria”

Approximately, 2 g of the studied Cyanobacterial mats were collected and preserved in Eppendorf tubes containing RNAlater to maintain RNA integrity. Samples were transported to the laboratory and processed immediately upon arrival. For the morphological analysis, the cyanobacterial mats were examined and microphotographed using the LEICA LAS version 4.12.0 image analysis software (Leica Microsystems Limited and CMS GmbH, Switzerland).

### 2.2 DNA extraction

Total DNA was extracted from the samples by SGS-Global Biosciences Center (Portugal), using DNeasy PowerSoil Pro Kit, following internally optimized procedures, including quality control, PCR amplification, and library preparation steps. Following internally optimized procedures, the 16S rRNA gene was prepared for each sample along with negative and positive laboratory controls, according to internally optimized procedures. All samples meeting the established quality control thresholds were selected for further processing.

### 2.3 Amplicon sequencing

The primer set 338F (5′-ACTCCTACGGGAGGAGGCAGCAG-3′) and 806R (5′ GGACTACHVGGGGTWTCTAAT-3′), tagged with Illumina adapters and linker sequences were used to target the V3-V4 regions of the bacterial 16S rRNA gene. Libraries were sequenced on Illumina NextSeq 2000 platform in 2×300 mode. The sequencing run performance was within the platform specifications. The 16S amplicon sequencing data for this study have been deposited in the European Nucleotide Archive (ENA) at EMBL-EBI under accession number PRJEB88730.

### 2.4 Microbial community structure and functional analysis of bacteria

16S rRNA gene raw sequences from Illumina NextSeq 2000 were pre-processed by removing primers and adapters using QIIME2 platform. Quality filtering, trimming, error models, and dereplication were performed with default settings. Trimmed sequences were denoised to ASV using the dada2 R package. Subsequently, taxonomy at different taxonomic rank levels was assigned using the Naïve Bayes classifier based on SILVA database version 138-NR99.^1^

Differences in community composition from freshwater and saline lakes were analyzed using linear discriminant analysis effect size (LEfSe) using a standard threshold of 2.0 logarithmic LDA score and an alpha value of 0.05 for Kruskal-Wallis and paired Wilcoxon test. The core microbiome was assessed using an abundance and occupancy-based method. Briefly, abundance data was transformed into relative abundances via compositional normalization. The definition of core microbiome followed standard thresholds of detection and prevalence, based on the approach outlined by Salonen et al. (2012). Specifically, ASVs were considered part of the core if they were detected at a minimum relative abundance of 0.1% in at least 95% of the samples. The functional analysis was performed using the FAPROTAX software (Louca et al., 2016).

### 2.5 Phylogenetic analysis of Cyanophyceae

To determine the phylogenetic position of Cyanophyceae sequences from the analyzed samples and obtain a more precise identification at the genus level, the 16S rRNA gene sequences were aligned to those of reference strains of 19 Cyanophyceae orders, according to Strunecký et al. (2023). Alignment was performed using ClustalW, in MEGA11: Molecular Evolutionary Genetics Analysis version 11 (Tamura et al., 2021). The final dataset, comprising Cyanophyceae sequences from this study along with the reference strains, consisted of a total of 520 sequences. The phylogenetic tree was built using maximum likelihood analysis. GTR + G + I evolutionary model was selected by MEGA 11. The robustness of the ML tree was estimated by bootstrap percentages, using 1,000 replications using IQ-Tree online version v1.6.12 (Trifinopoulos et al., 2016). The tree was edited using ITOL.^2^ For the identification of taxa, beyond the phylogeny, 16S rRNA gene identity (p-distance) was used, and sequences were considered to belong to the same genus when presenting >95% identity (Komárek et al., 2014).

### 2.6 Statistical analysis

Before conducting diversity analyses, samples were rarefied to the same sequencing depth using the R package Microeco (version 1.9.0) (Liu et al., 2021). Taxonomic diversity of the microbiome across both lagoon groups was estimated using the Chao1 and Shannon indices, with analyses performed using the R package vegan (Oksanen et al., 2025). Normality and homogeneity of variances were assessed using the Shapiro–Wilk and Levene’s tests, respectively. When these assumptions were met, analysis of variance (ANOVA) was applied; otherwise, non-parametric comparisons were conducted using the Mann–Whitney U test. All statistical analyses were performed using R version 4.3.2 (R Core Team, 2020, Vienna, Austria). Differences were considered statistically significant at *p* < 0.05.

## 3 Results

### 3.1 Morphological diversity

The morphological examination of the cyanobacterial mat samples revealed the presence of seven different morphotypes, predominantly dominated by *Nostoc* Bornet & Flahault, followed by heterocytous and homocytous filamentous forms.

**Figure 2:**
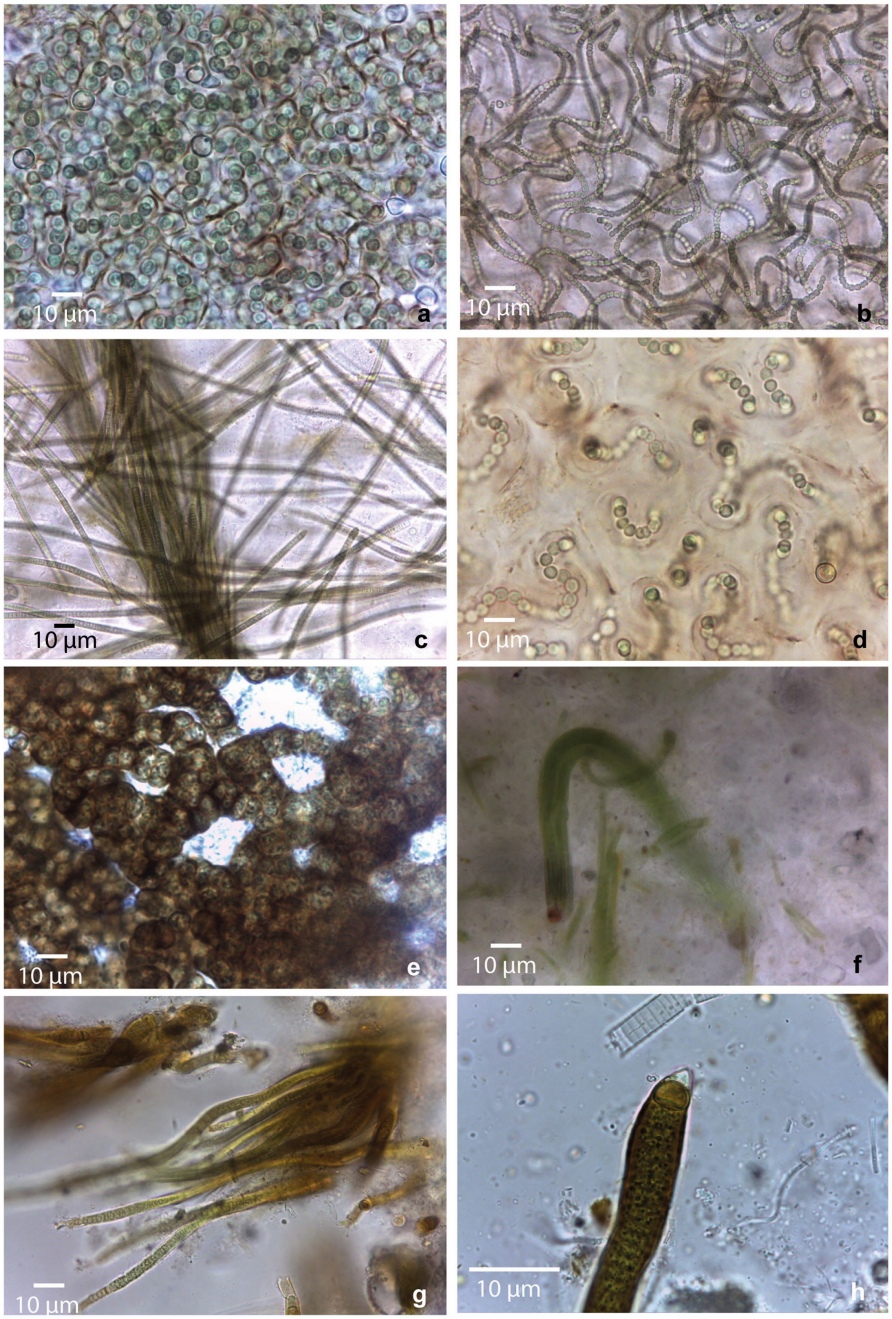
Morphological diversity of cyanobacterial morphotypes observed in collected mats. **(a)** *Nostoc* sp1; **(b)** *Nostoc* sp2.; **(c)** *Microcoleus* sp.; **(d)** *Nostoc* sp3.; **(e)** *Nostoc* sp4.; **(f)** Coleofasciculales sp.; **(g,h)** Rivulariaceae sp.

Samples from saline environment of Salar de Uyuni (GBC221, 222), were composed by two different *Nostoc* morphotypes. In GBC221, trichomes were short, densely arranged, and enveloped by individual mucilaginous sheaths in addition to the colony envelope (Figure 2a). Conversely, in GBC222, trichomes were longer, loosely entangled, and lacked individual sheaths (Figure 2b).

Regarding the freshwater stream samples, in the Catal lake (GCB223) sample, a *Nostoc* morphotype identical to GBC222 was observed. In the freshwater stream adjacent to Laguna Colorada (GBC224), a homocytous filamentous morphotype was identified. This morphotype presented cylindrical trichomes of 5–6 μm, commonly attenuated toward the ends, within thin colorless sheaths, sometimes forming fascicles and cells shorter than wide. These characters are similar to *Microcoleus* Gomont and *Phormidium*-like types (Figure 2c).

In the stream close to Laguna Chalviri (GBC225) a third *Nostoc* morphotype, resembling that of GBC221, but with less densely arranged trichomes within the colony, was observed (Figure 2d).

The single sample collected from the shoreline of the freshwater Laguna Chojllas Pequeña (GBC226), was composed of a fourth *Nostoc* morphotype, distinguished by a brownish envelope and denser arrangement of trichomes, compared to the other samples (Figure 2e).

*Nostoc* morphotypes identified in samples from the shores of saline lakes Laguna Coruto (GBC227), and Laguna Pastos Grandes (GBC229) were identical to those observed in samples GBC226 (freshwater) and GBC221 (saline), respectively. Thus, this suggests a remarkable ecological plasticity within the genus, allowing it to thrive in both freshwater and saline. Sample GBC228 exclusively featured a homocytous morphotype, with parallel arrangement of the trichomes forming fascicles. Trichomes appeared constricted, cylindrical, 4–6 μm wide, with cells longer than wide, and conical apical cells. These characteristics resemble those of the Coleofasciculales (Figure 2f). Samples GBC230 and GBC231 from Laguna Pastos Grandes consisted of a heterocytous filamentous morphotype with parallel arrangement of trichomes, characterized by heteropolar (basal and intercalar heterocytes), attenuated toward the ends, and thick yellow-brownish sheaths. Vegetative cells were shorter than wide, features typical of the Rivulariaceae family (Figures 2g,h).

### 3.2 16S rRNA gene analysis of the Cyanophyceae biodiversity and abundance

Amplicon sequencing produced 4,113 16S rRNA gene amplicon sequence variants (ASVs) for the bacterial community. Of these, 310 ASVs were identified as Cyanobacteria, with 134 classified as Cyanophyceae (Strunecký et al., 2023) using a Naïve Bayes Classifier trained on 16S data from the SILVA database. This metabarcoding analysis identified 32 different taxa (Supplementary Table S1). Based on the number of reads, the relative abundance of the class Cyanophyceae was 20%, with bacteria from other classes constituting the remaining 80% of bacterial abundance.

**Figure 3:**
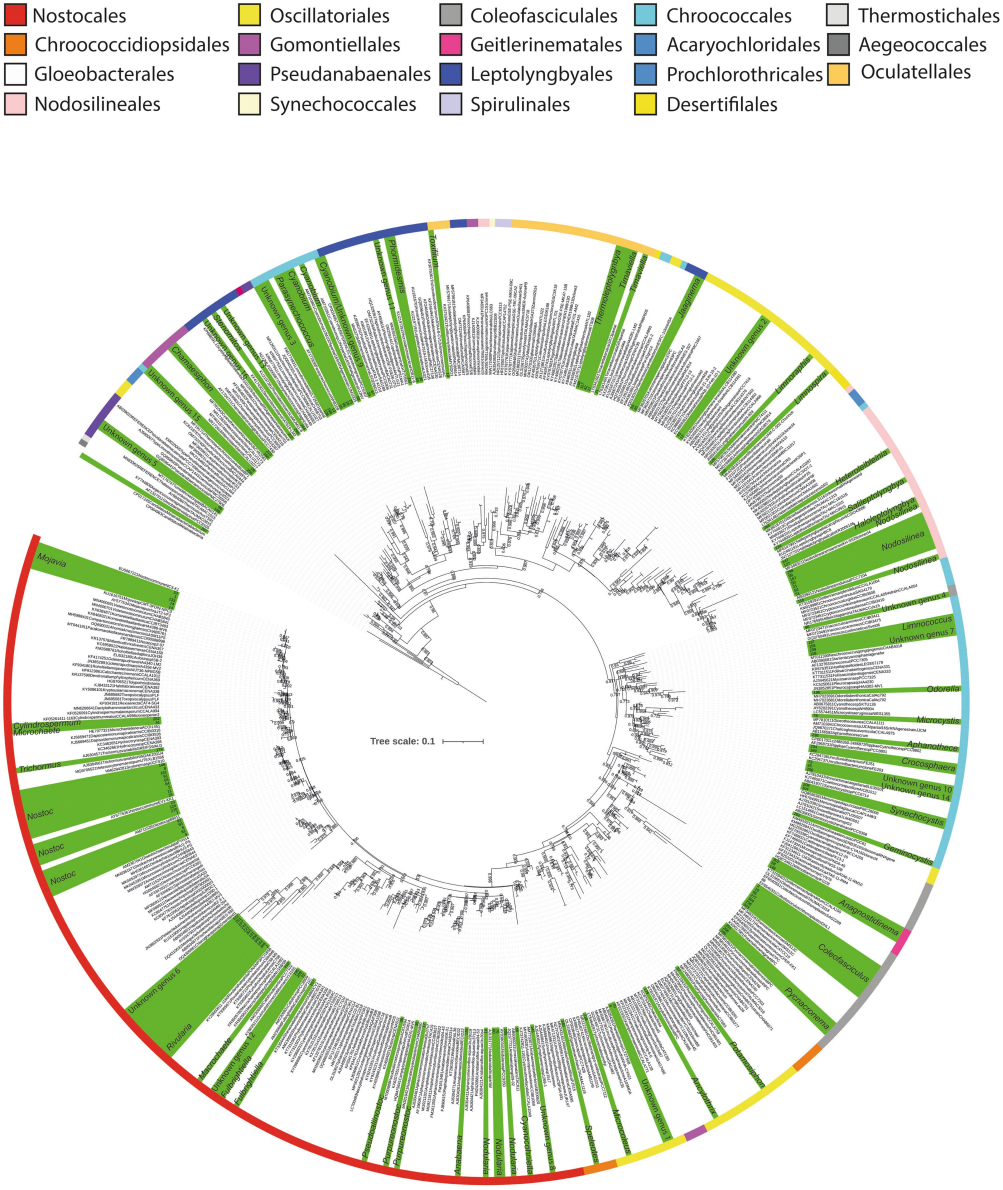
Maximum likelihood phylogenetic tree based on 16S rRNA sequences of the Cyanophyceae. Sequences obtained in this study are highlighted in green, and identified at genus level. Labels corresponding to our sequences are represented by numbers. Colored stripes indicate the different orders within Cyanophyceae.

Regarding Cyanophyceae sequences, additional qualitative analyses were conducted to achieve a more precise identification of the cyanobacterial genera present in the samples. For this purpose, a 16S rRNA gene ML phylogenetic analysis (Figure 3) and 16S identity (p-distance) comparisons were performed. These analyses are considered more accurate than SILVA-based metabarcoding approach, as the reference strains included in the phylogenetic tree were manually curated by the authors, ensuring a more precise and reliable taxonomic classification. Supplementary Table S2 provides detailed identification of the Cyanophyceae sequences at the genus level, including the 16S rRNA gene identity (*p-*distance) with the closest phylogenetically related cyanobacterial genus.

These analyses revealed that among the 134 Cyanophyceae ASVs, 104 could be identified within forty-two different genera, distributed across nine orders. This represents a significantly greater diversity than the morphological analysis, which identified only seven morphotypes predominantly related to *Nostoc*. Furthermore, the phylogenetic analysis detected an even higher level of diversity compared to the metabarcoding analysis, which identified 32 taxa (Figure 3; Table 1; Supplementary Tables S1, S2).

Comparing genera, according to the phylogenetic analysis, a total of 18 different ASVs were identified as *Nostoc,* making it the genus with the highest diversity of sequences. Additionally, nine different ASVs were within the *Nodosilinea* clade, while eight different sequences were identified as *Coleofasciculus* Siegesmund et al. The genera *Cyanobium* Rippka & Cohen-Bazire and *Mojavia* Reháková & Johansen, exhibited five ASVs each (Figure 3, Supplementary Table S2).

The order with the highest diversity of genera was Nostocales, encompassing a total of 11 genera, followed by Chroococcales, with nine, and Oscillatoriales with five (Supplementary Table S2).

In addition to these identified sequences, 30 additional sequences were distributed among 16 distinct monophyletic clades. Interestingly, none of these sequences were closely related to any previously described cyanobacterial genera, exhibiting less than 95% 16S rRNA gene identity with their closest phylogenetic relatives (Figure 3; Supplementary Table S2). These findings suggest the potential existence of at least 16 novel cyanobacterial clades that may warrant formal classification in the future.

Among these clades, the orders with the highest number of unidentified clades were Leptolyngbyales and Chroococcales, with four clades each, followed by Nostocales with three potential new genera. Primarily, these unidentified sequences have originated from the saline lake at Laguna Pastos Grandes (GBC231) and a freshwater stream at Laguna Chalviri (GBC225) (Figure 3).

Regarding the abundance of taxa (number of reads), the limitations of the Naïve Bayes Classifier in identifying taxa at the genus level prevented precise determination of the number of reads for each genus identified by the phylogenetic analysis. Alternatively, the number of reads for “genera complexes” were identified as shown in Supplementary Table S1. Through this approach, the most abundant complex was found to be *Mojavia*/*Nostoc*/“unknown genus 6,” with 90,781 reads. This complex was detected in Salar de Uyuni (GBC221, GBC222), Catal Lake (GBC223), Laguna Chalviri (GBC225, GBC226), Laguna Coruto (GBC227), and Laguna Pastos Grandes (GBC229), indicating a remarkable ability of these genera to adapt to various conditions, ranging from freshwater to saline environments. Notably, this complex represented 57% of the total Cyanophyceae reads across all samples. *Nostoc*-like macroscopic colonies were commonly found thriving in these extreme environments.

The second most abundant genus complex was *Coleofasciculus/Pycnacronema* Martins & Branco, with 21,366 reads, representing 13% of the total Cyanophyceae across all samples. However, this complex was found exclusively at Laguna Mama Khumu (GBC228), a saline lake (Table 1; Supplementary Table S1). The *Coleofasciculus/Pycnacronema* mats were observed growing beneath a thin layer of pink bacterial biofilm on soil.

The third and fourth most abundant genera complexes were *Rivularia* Bornet & Flahault and “unknown genus 1”/*Limnoraphis/Microcoleus/Salileptolyngbya*, with 19,766 and 17,452 reads, respectively. *Rivularia* accounted for 12.5% of the total Cyanophyceae reads across all samples. It was identified in Laguna Pastos Grandes (GBC229, GBC230, GBC231) and Laguna Chalviri (GBC225), showcasing its adaptability to both saline and freshwater environments. The complex “unknown genus 1/*Limnoraphis/Microcoleus/Salileptolyngbya* Zhou & Ling comprised 11% of the total Cyanophyceae reads across all samples and was exclusively sampled from a freshwater stream at Laguna Colorada (GBC224). Additionally, notable findings include *Prochlorococcus* Chisholm et al., *Timaviella* Sciuto & Moro, *Thermoleptolyngbya* Sciuto & Moro and *Altericista* Averina et al., which are further discussed in the subsequent sections.

Among the samples analyzed, the Laguna Chalviri stream (GBC225) stood out as the most diverse, with 17 taxa identified by metabarcoding, corresponding to 22 genera identified by phylogenetic analysis (Table 1; Supplementary Table S1). Regarding Cyanophyceae abundance across samples, GBC226, collected from the freshwater of Laguna Chojllas, exhibited the highest number of Cyanophyceae reads (26,284), constituting 16.6% of the total reads. Despite its high number of reads, this sample showed low diversity, mainly composed of the *Mojavia*/*Nostoc*/“unknown genus 6” complex. Similarly, sample GBC228, from a saline lake in Laguna Mama Khumu, contained 21,719 reads, representing 13.7% of the total reads, with *Jaaginema* Anagnostidis & Komárek and the *Coleofasciculus*/*Pycnacronema* complex as the dominant taxa. The third sample with the highest number of reads was Laguna Chalviri (GBC225) with 18,230 reads, representing 11.5% of the total reads. Notably, as mentioned above, this sample had the highest diversity, comprising 17 different genera complexes (Table 1; Supplementary Table S1).

### 3.3 Cyanophyceae and the associated microbiome

Metabarcoding analysis revealed a dominance of organisms classified into six major phyla: Actinobacteriota, Bacteroidota, Cyanobacteria, Firmicutes, Proteobacteria, and Verrucomicrobiota (Supplementary Figure S1; Supplementary Table S3). Interestingly, Campilobacterota, Deinococcota, and Halanaerobiaeota appeared to be exclusively represented in the saline samples. Regarding Catal Lake, despite being a freshwater lagoon, this sample exhibited unusually high bacterial viability compared to other freshwater systems (Supplementary Figures S1–S4). Due to this atypical profile, we excluded it from group-level comparisons of bacterial diversity and community composition between freshwater and saline lakes. Instead, the Catal Lake sample was considered separately and used as a reference to provide contextual insight, rather than being included in the comparative statistical analyses.

**Figure 4:**
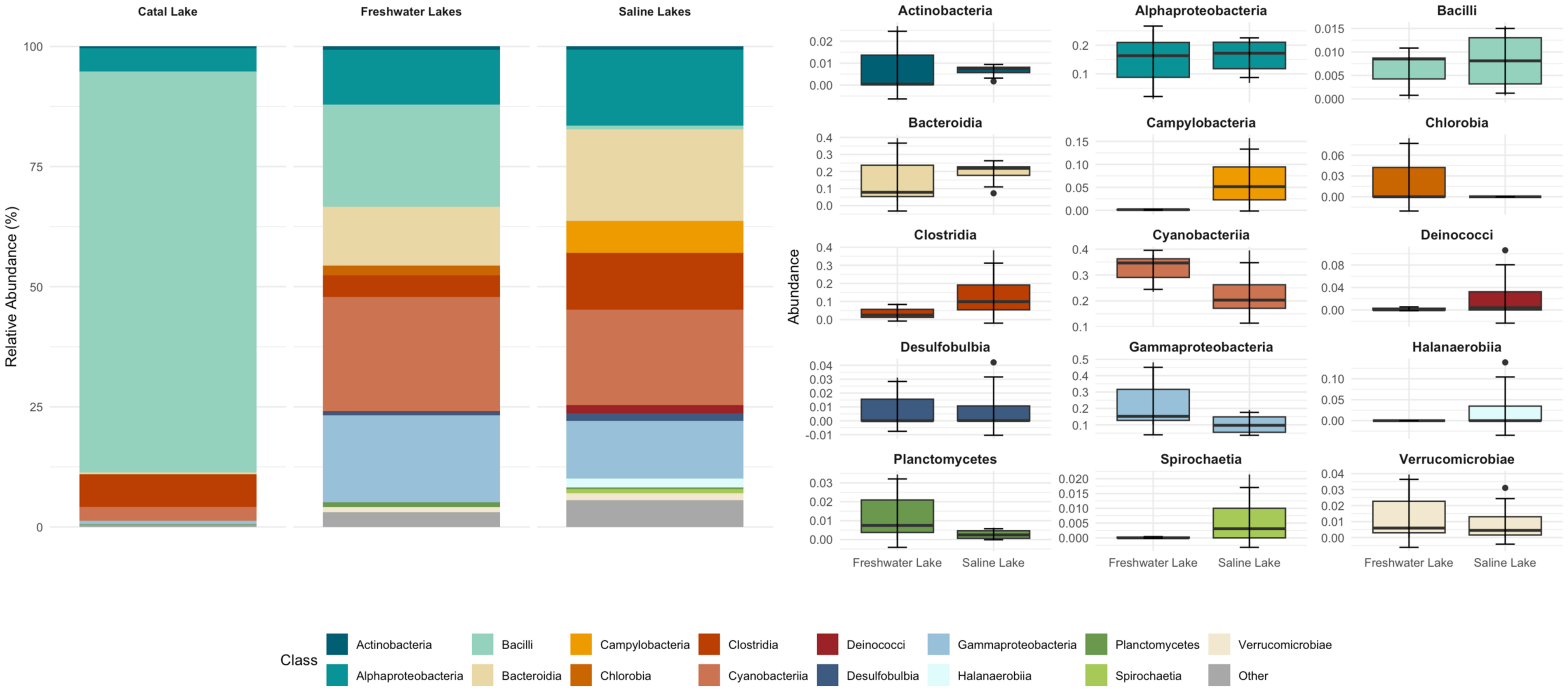
Relative abundance of bacterial taxa at the class level across lacustrine environments. The stacked bar plot (left) shows the mean relative abundance of dominant bacterial classes identified in three aquatic systems: freshwater lakes, saline lakes, and the unique Catal Lake. Classes with low relative abundance are grouped as “Other.” The boxplots (right) illustrate the distribution of abundances, with each boxplot representing the interquartile range, the line indicating the median, and whiskers extending to 1.5 times the IQR; outliers are shown as individual points. For grouped comparisons, samples from Pastos Grandes and Uyuni lakes were averaged per site, as no significant differences were detected between them.

**Figure 5:**
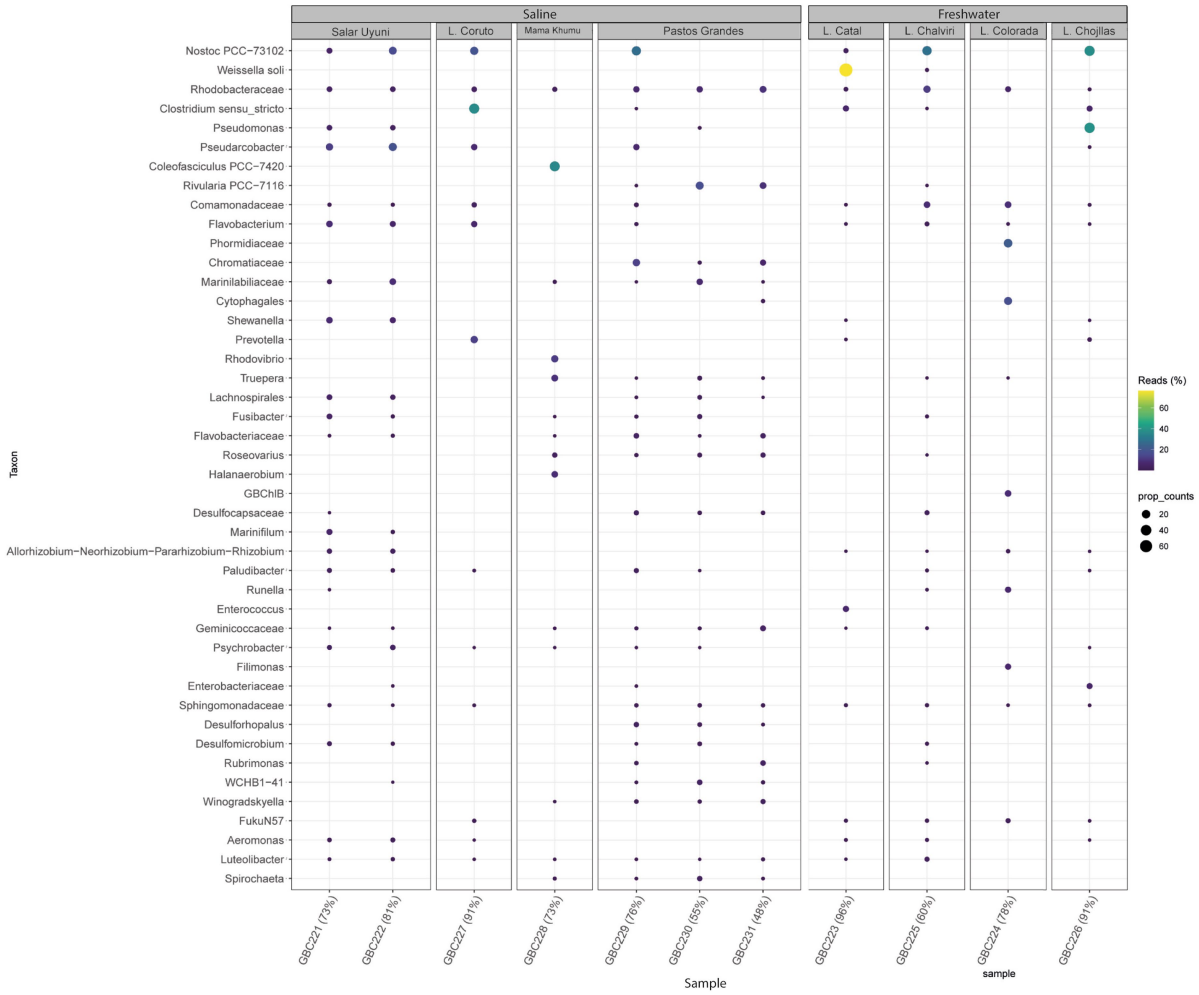
Bubble map showing the most abundant bacteria genera detected across all samples and illustrating microbial community variations among samples, according to metabarcoding analysis.

At the class level, Alphaproteobacteria, Bacteroidia, Gammaproteobacteria, Clostridia, Cyanophyceae, and Campylobacteria were dominant across both sample groups. The bacterial composition of the different samples is illustrated in Figures 4, 5.

Most bacterial groups were present across all samples, although their relative abundances varied. The core microbiome consisted of bacteria from the classes: Acidomicrobia, Actinobacteria, Bacteroidia, Cyanobacteria, Deinococci, Clostridia, Gammatimonadetes, Planctomycetes, Alphaproteobacteria, Gammaproteobacteria, and Verrucomicrobiae.

Samples collected from freshwater reservoirs exhibited the highest bacterial diversity, with Alphaproteobacteria, Bacteroidia, Gammaproteobacteria, Cyanobacteria, and Bacilli being the dominant classes. In contrast, samples from the saline lakes were predominantly composed of Alphaproteobacteria, Bacteroidia, Gammaproteobacteria, Cyanobacteria, and Clostridia (Figure 4).

Figure 5 illustrates the most abundant bacterial genera identified through metabarcoding. *Nostoc* was prevalent and abundant across all environments. *Pseudomonas* showed higher prevalence in Laguna Chojllas, particularly associated with *Mojavia, Nostoc*, “unknown genus 6” and *Chamaesiphon* Braun, but was scarce in other samples. *Weissella soli* Magnusson was found in high abundance in Catal Lake, but was absent from the remaining sites. *Clostridium* Cohn was notably abundant in Laguna Coruto.

**Figure 6:**
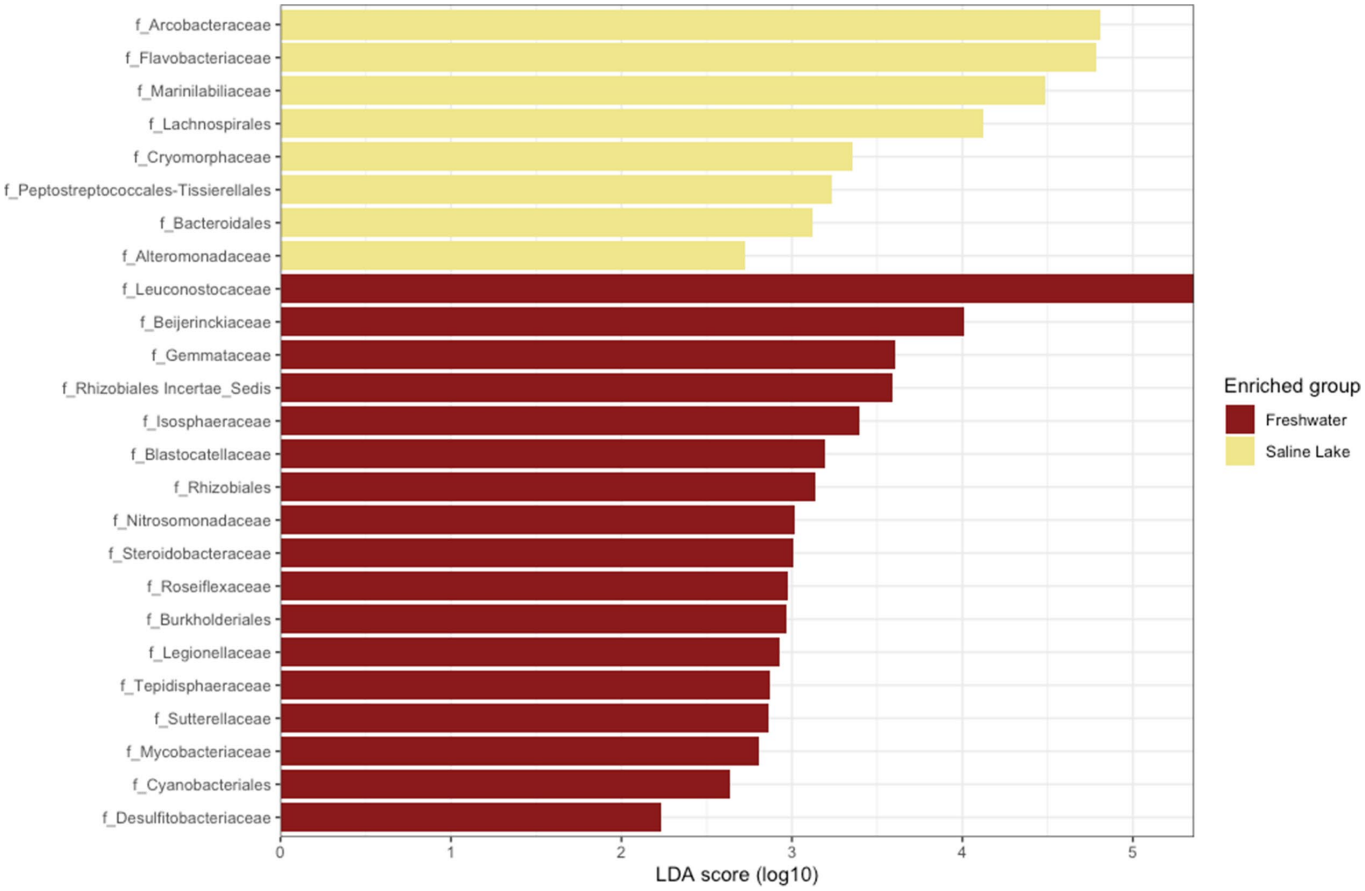
Linear discriminant effect size analysis (LEfSe) results for freshwater and saline lakes. Taxonomic groups are shown based on statistical significance (Kruskal–Wallis and Wilcoxon tests, alpha = 0.05) and LDA score > 2.0.

When analyzing potential biomarkers for freshwater and saline environments, a differential community composition was detected at all taxonomic levels, including at the class level. Campylobacteria was identified as a biomarker for saline environments, while Blastocatellia appeared to be exclusive to freshwater streams (Supplementary Figure S5). At the family level, eight markers were identified for saline environments and 17 for freshwater streams. As shown in Figure 6, Arcobacteriaceae and Flavobacteria were the most representative families in saline samples, whereas Leuconostocaceae and Beijerinckiaceae were dominant in freshwater streams.

Regarding the relationship between cyanobacterial mats and their respective microbiome, the microbial communities associated with *Nostoc* mats exhibited significant diversity across different environments (Table 1; Supplementary Figure S4). For example, in Salar de Uyuni (samples GBC221 and GBC222), the *Nostoc*-associated microbiome comprised similar proportions of Alphaproteobacteria, Bacteroidia, Gammaproteobacteria, Clostridia, Bacilli, and Campylobacteria. In contrast, in Catal Lake (GBC223), the *Nostoc* microbiome was predominantly composed of *Weissella* (Bacilli). In Laguna Chojllas Pequeña (GBC226), *Pseudomonas* (Gammaproteobacteria) dominated the microbial community associated with *Nostoc* mats, whereas in Laguna Coruto (GBC227) *Clostridium* (Clostridia) was the dominant genus (Table 1; Supplementary Figure S4). These results suggest that the composition of the microbiome associated with *Nostoc* mats is likely more influenced by environmental factors than by the *Nostoc* mat itself.

When comparing samples from freshwater and saline environments, the microbiome associated with cyanobacterial mats showed strong variation driven by abiotic factors. Taken together, these findings underscore the likelihood that the structure of cyanobacteria-associated microbiomes is more regulated by the environmental conditions than by cyanobacterial identity alone.

**Figure 7:**
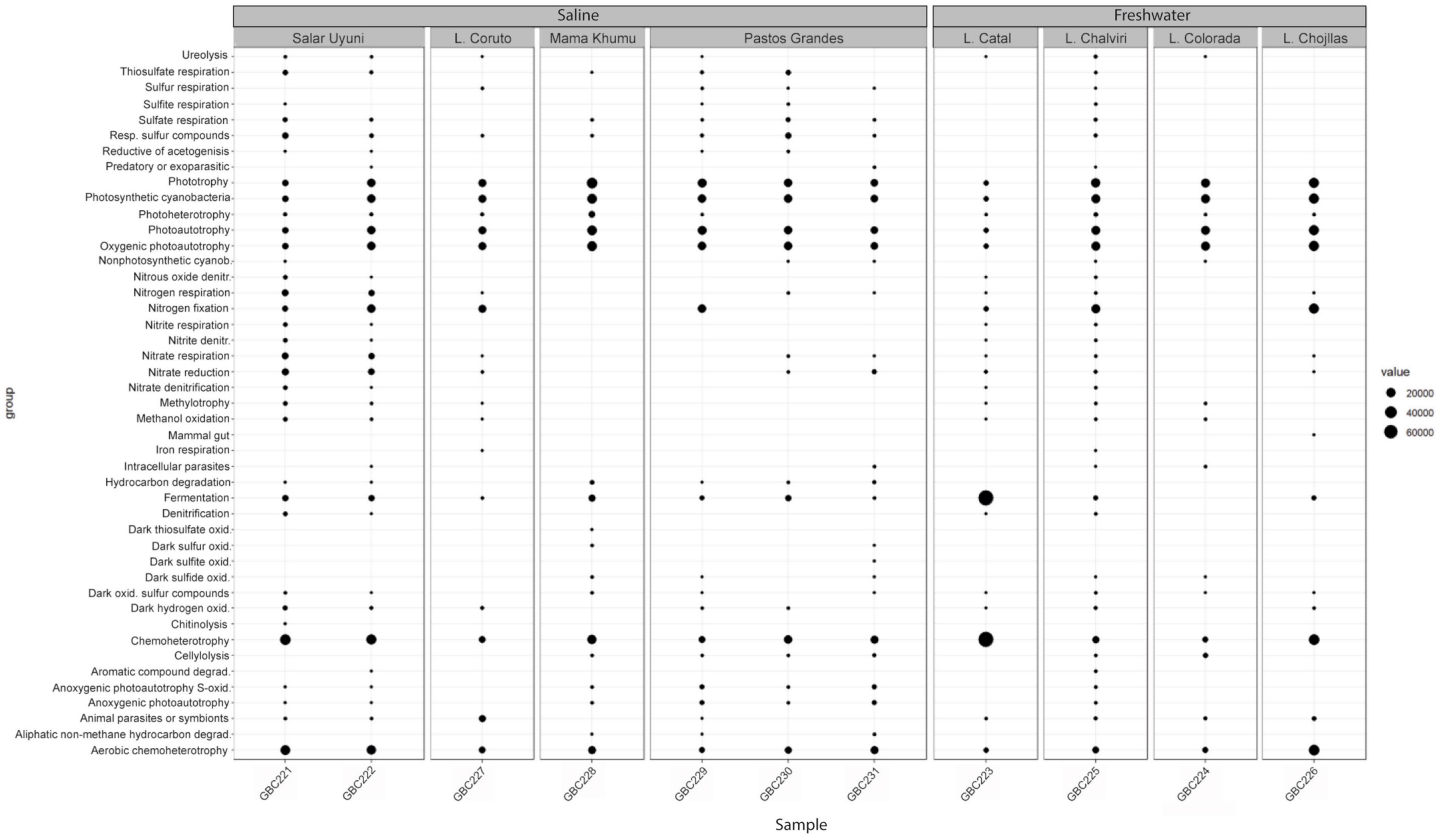
Functional profiles of the bacterial communities associated with cyanobacterial mats.

**Figure 8:**
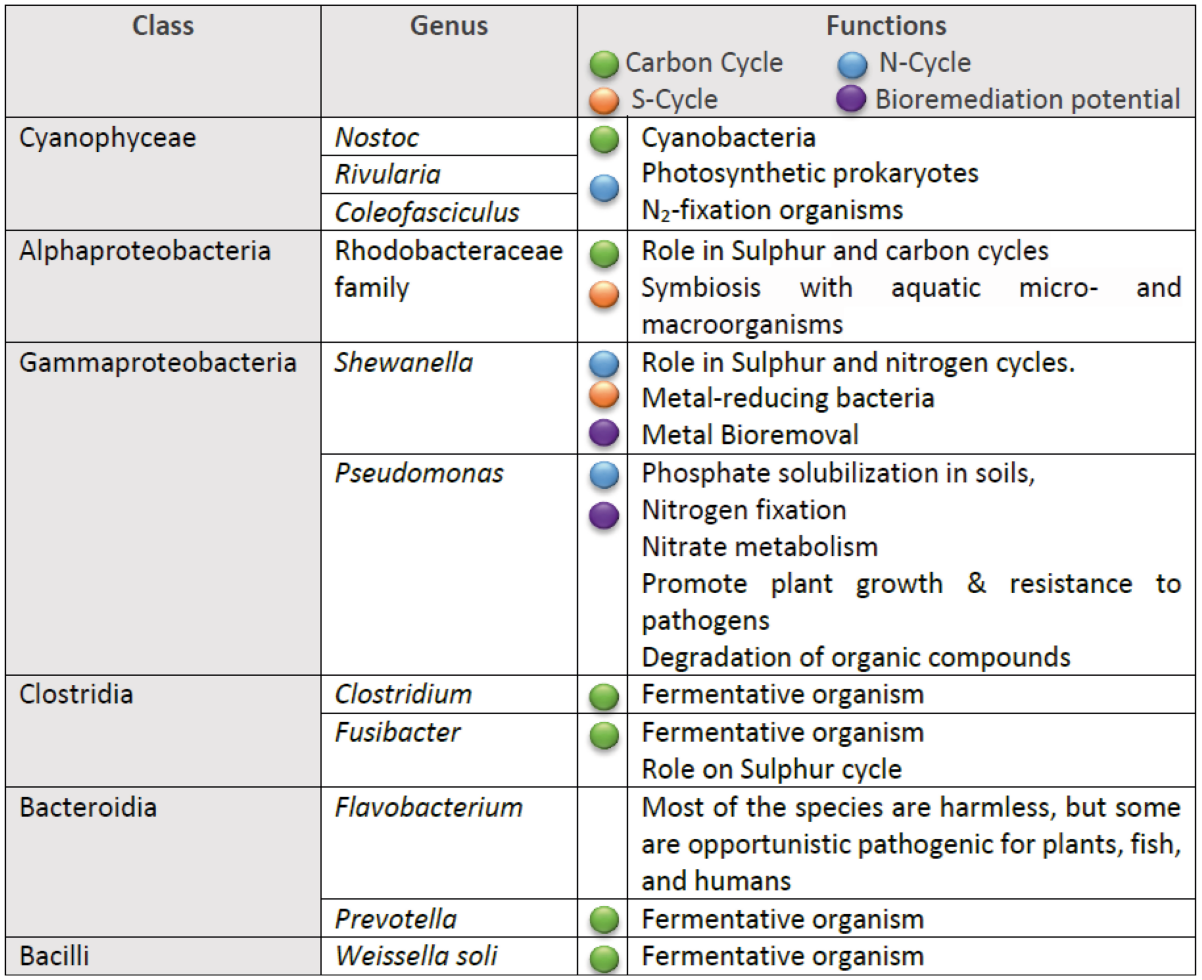
Summary of the metabolic traits of the most prevalent bacterial genera identified in the microbial communities.

The inferred metabolic functions of the associated microbiome are presented in Figure 7, while Figure 8 provides a more detailed description of the metabolic characteristics of the most relevant microorganisms. Overall, the major functions of the microbiome encompassed phototrophic and chemoheterotrophic metabolisms, sulfur respiration, nitrogen fixation, nitrate and nitrite metabolism, and fermentation. Notably, nitrogen-fixing cyanobacteria, such as *Nostoc*, *Rivularia,* and *Coleofasciculus* were prevalent, along with bacteria known for their bioremediation potential, including members of the genera *Shewanella* and *Pseudomonas*.

## 4 Discussion

Morphological and the 16S rRNA gene molecular analyses of Cyanophyceae were generally consistent, although the molecular analysis unveiled a broader diversity at the genus level, due to its higher sensitivity. Additionally, we successfully correlated the morphological findings with the phylogenetic tree. For example, the phylogenetic analysis confirmed that 18 ASVs obtained from the *Nostoc* mats, from samples GBC221, 222, 223, 225, 226, 227 and 229, indeed belonged to this genus. *Nostoc* also accounted for the majority of 16S rRNA gene sequences identified in those samples (Supplementary Table S1).

Morphologically and ecologically, our *Nostoc* morphotypes perfectly fit in the description of this genus. The genus typically thrives in terrestrial environments, especially on wet sandy soils or semideserts (Komárek, 2013). While the genus is cosmopolitan, with some species being predominantly terrestrial, others are known to inhabit in a wide range of aquatic ecosystems. Moreover, certain species exhibit an affinity for extreme environments (Dodds et al., 1995; Aguilar et al., 2019). Our morphological and habitat analysis were in agreement with those characters and conditions. This underscores the high ecological adaptability of this genus, as it was evidenced by its ubiquitous presence in both freshwater and saline samples of our work.

Combined with these *Nostoc* mats, we also found 16S rRNA gene sequences of *Mojavia*, a *Nostoc*-like genus, characterized by the lack of macroscopic thalli. It had only been observed in cultured isolates and currently encompasses three recognized species. Its occurrence has been exclusively observed in desert soils (Johansen et al., 2007; Baldarelli et al., 2022). However, in our study, the genus was found particularly in the sample GBC228, collected from the shore of the saline Laguna Mama Khumu—a previously undocumented habitat for the genus. This unique new ecological niche, suggests the presence of a potential novel *Mojavia* genus. Nevertheless, confirmation of this hypothesis requires the isolation of a strain from the sample and longer 16S rRNA gene sequences for a more robust phylogenetic analysis.

The morphotype identified as Coleofasciculales, collected from L. Mama Khumu (GBC228), was confirmed as phylogenetically related to *Coleofasciculus* and *Pycnacronema* (Figure 2b; Supplementary Table S2). This was evidenced considering that from this sample we obtained three ASVs identified as *Pycnacronema* and seven identified as *Coleofasciculus*. These sequences collectively represented nearly 100% of the Cyanophyceae reads in this sample. *Coleofasciculus* and *Pycnacronema* are both in the Coleofasciculales order (Strunecký et al., 2023), and are distinguished from each other primarily by the presence of multiple trichomes per sheath in *Coleofasciculus*, whereas *Pycnacronema* presents a single trichome per sheath. Ecologically, *Pycnacronema* is terrestrial, while *Coleofasciculus* inhabits primarily the benthic marine zone (Siegesmund et al., 2008; Martins et al., 2019). The populations found by our study exhibited morphological similarities to *Pycnacronema* and ecological affinities with both genera, given that GBC228 was collected from the shore of a saline lake (terrestrial). Again, to achieve a precise identification of our populations, the isolation of strains is necessary to obtain 16S rRNA gene sequences for further analysis and classification.

The heterocytous filamentous morphotype observed in samples GBC230 and GBC231 shared morphological similarities with *Streptostemon* and *Dapisostemon* (Sant’Anna et al., 2010; Hentschke et al., 2016) particularly in terms of the basal positions of heterocyte and the formation of fascicles. However, our populations were distinguished from them by the presence of attenuated trichomes—a common feature among Rivulariaceae family. Phylogenetic analysis supported this classification, revealing that five ASVs from our populations were closely related to *Rivularia*. This genus is typically found in marine environments, and is characterized by the presence of hair-like structures at the ends (Komárek, 2013). Although our populations lacked this hair-like structures, they are phylogenetically clustered with the reference strain *Rivularia halophila* PUNANP3, sharing more than 97% of 16S rRNA identity with it (Figure 2a; Supplementary Table S2), therefore indicating their accurate classification within this genus.

The detection of *Prochlorococcus*, *Timaviella, Thermoleptolyngbya*, *Altericista* in the collected samples also represents notable findings. For instance, *Prochlorococcus* is typically recognized as a picoplanktonic genus found in marine environments (Komárek, 2013). However, in our study it was identified in samples collected from a freshwater stream. This finding suggests ecological plasticity within *Prochlorococcus*, but further studies involving the isolation of strains from these environments are necessary to confirm their adaptability.

The presence of *Timaviella*, a cyanobacterial genus, in the analyzed samples is particularly intriguing given its original description from Greek caves, and more recent discovery in rock walls in USA and sand dunes in Baltic Sea (Sciuto et al., 2017; Mai et al., 2018; Mikhailyuk et al., 2022). The dissimilarity between these environments and the sample sites included in our study raises the possibility that the Bolivian population is a new species. To further assess this possibility, additional studies should be performed.

The identification of *Thermoleptolyngbya* populations from freshwater samples in Bolivia is noteworthy, considering their origin in geothermal habitats across Europe and the USA (Sciuto and Moro, 2016). Similarly, to what was observed with *Timaviella*, this finding raises suspicion that the Bolivian population may represent a new species of the genus. A similar scenario is observed with our *Altericista* population. Up to now, the genus presents only one species, which is documented from Russia (Averina et al., 2021). These observations support the hypothesis that these populations represent new taxa.

Considering the abundance of sequences, the Cyanophyceae group was responsible for 20% of the number or reads across the whole microbiome. We considered it a high proportion, since other authors have found 4.7 to 5.6% of cyanobacteria relative abundance in microbiomes (Miralles et al., 2023). However, this high proportion in our study was expected, given that our sampling targeted cyanobacterial macroscopic mats. Additionally, the high relative abundance of *Nostoc* sequences (57%) was also expected, considering that macrocolonies of this genus were very common in the field across all types of environments.

Regarding the Cyanophyceae-associated microbiomes, various species of *Nostoc*, such as *Nostoc flagelliforme* and *Nostoc commune* have been described to form associations with heterotrophic bacteria (Graham et al., 2014; Han et al., 2015; Inthasotti and Pathom-aree, 2015). The pioneer study on microbiome inside *Nostoc* macroscopic colonies was conducted by Aguilar et al. (2019), revealing a distinct microbial community within *Nostoc* colonies, contrasting with the microbial composition of the surrounding environment. Our findings are consistent with these studies, as we also identified Alphaproteobacteria, Bacteroidia, Clostridia, and Gammaproteobacteria in our *Nostoc* samples. However, upon comparison of our *Nostoc* samples, we found significant differences in the composition and proportion of the associated microbiome among them. This dissimilarity suggests that environmental conditions may influence microbiome composition. Moreover, Inthasotti and Pathom-aree (2015) investigated the microbiome growing on *Nostoc* colonies and found a rich community of Actinobacteria, which were not observed in our study. This report along with our findings indicate that the *Nostoc-*associated microbiome composition is probably more influenced by the surrounding environment than the cyanobacterial mat. This hypothesis is further supported by the distinct microbial composition found when comparing the two samples containing the heterocytous fasciculate morphotype “Rivulariaceae” (GBC230 and GBC231). To confirm this, comparative studies of mats and their surrounding environments are needed.

Regarding the functional analysis, although FAPROTAX has certain limitations—such as generalization within taxa, lack of information on gene expression, and reliance on known taxonomic assignments—it nevertheless enabled the identification of a broad spectrum of microbial functions. These findings highlight the vast and largely untapped biotechnological potential of Bolivia’s extreme environments. For instance, phototrophic organisms offer promising applications in biofuel production, carbon sequestration (Love, 2022), and the biosynthesis of value-added bioproducts such as bioplastics (Souza and Gupta, 2024), contributing to strategies for mitigating anthropogenic climate change. Chemoheterotrophic bacteria are valuable for bioremediation, including the degradation of toxic pollutants such as oil-derived waste (Kämpfer et al., 1993), and for the production of antibiotics (Procópio et al., 2012) and industrial enzymes (Danilova and Shaipova, 2020). Sulfur-respiring microorganisms show potential for the bioremediation of heavy metals and other toxic compounds, particularly in industrially contaminated environments (Chen et al., 2025). Furthermore, nitrogen-fixing cyanobacteria contribute to sustainable agriculture by enhancing soil fertility through the natural addition of bioavailable nitrogen, thereby reducing dependence on synthetic fertilizers (Joshi et al., 2024; Singh et al., 2016).

Finally, our study represents the first comprehensive metabarcoding analysis of cyanobacterial mats from Bolivia, including their associated microbiomes. It is also the first to integrate multiple complementary approaches: high-throughput metabarcoding, morphological characterization, 16S rRNA gene phylogeny using curated reference strains, pairwise 16S rRNA identity (p-distance) analysis, and genomic functional analysis. Although previous studies have already reported microbial diversity in the Bolivian Altiplano using metabarcoding, our study offers a more integrative framework. For instance, Daga-Quisbert et al. (2023) found abundant populations of the cyanobacterial genus *Microcoleus* and members of the order Pseudomonadales in Laguna Pastos Grandes, which aligns with our findings. These authors also documented the presence of Burkholderiales and Halobacteriales. Similarly, Pérez-Fernandez et al. (2019) found many unclassified members of the Gammaproteobacteria in Salar de Uyuni, a group we also detected in this environment. These results, along with our findings, suggest that many new bacterial and cyanobacterial taxa remain to be described in these ecosystems. Moreover, our results also report an expansion in the distribution of numerous cyanobacterial genera.

## Data Availability

The datasets presented in this study can be found in online repositories. The names of the repository/repositories and accession number(s) can be found at: https://www.ebi.ac.uk/ena, PRJEB88730.

## Footnotes

^1^ https://www.arb-silva.de/

^2^ https://itol.embl.de/
