# Supplementary material for: Cyanobacterial mats and their associated microbiomes in saline and freshwater lakes from the Bolivian Altiplano
Source: Front Microbiol. 2025 Jul 23;16:1650455. doi: 10.3389/fmicb.2025.1650455 (PMC12325342; doi:10.3389/fmicb.2025.1650455)
Supplement: Supplementary file 1 [file Table_1.docx]

Table S1. Abundance (number of reads) of Cyanobacteria found in each sample

| Phylogenetic identification (Genera complexes) | Metabarcoding identification | GBC228 | GBC231 | GBC225 | GBC229 | GBC230 | GBC223 | GBC224 | GBC221 | GBC222 | GBC226 | GBC227 | Total reads | Rel.abundance across all samples (%) (Cyanophyceae community) |
| --- | --- | --- | --- | --- | --- | --- | --- | --- | --- | --- | --- | --- | --- | --- |
| *Coleofasciculus, Pycnacronema* | *Coleofasciculus* PCC-7420 | 21366 | 0 | 0 | 0 | 0 | 0 | 0 | 0 | 0 | 0 | 0 | 21366 | 13,51 |
| *Anagnostidinema* | *Geitlerinema* LD9 | 0 | 0 | 12 | 0 | 0 | 0 | 0 | 39 | 0 | 0 | 0 | 51 | 0,03 |
| Unknown genus 4 | Symphothece PCC-7002 | 0 | 20 | 0 | 0 | 0 | 0 | 0 | 0 | 0 | 0 | 0 | 20 | 0,01 |
| Unknown genus 10, Unknown genus 8 | Cyanobacterales | 0 | 0 | 100 | 0 | 0 | 12 | 0 | 0 | 0 | 0 | 0 | 112 | 0,07 |
| *Potamosiphon* | *Microseira* Carmichael-Alabama | 0 | 0 | 42 | 0 | 0 | 0 | 0 | 0 | 0 | 0 | 0 | 42 | 0,03 |
| *Limnococcus* | Chroococcales cyanobacterium – *Gloeocapsa* | 0 | 0 | 16 | 0 | 0 | 0 | 0 | 0 | 0 | 0 | 0 | 16 | 0,01 |
| Unknown genus 7 | *Gleocapsa* | 0 | 0 | 11 | 0 | 0 | 0 | 0 | 0 | 0 | 0 | 0 | 11 | 0,01 |
| *Crocosphaera*, Unknown genus 14 | Microcystaceae | 0 | 0 | 0 | 0 | 10 | 0 | 0 | 0 | 0 | 0 | 0 | 10 | 0,01 |
| *Microcystis* | *Microcystis* PCC-7914 | 0 | 0 | 26 | 0 | 0 | 0 | 0 | 0 | 0 | 0 | 0 | 26 | 0,02 |
| *Synechocystis* | *Synechocystis* PCC-6803 | 0 | 0 | 13 | 0 | 0 | 0 | 0 | 0 | 0 | 0 | 0 | 13 | 0,01 |
| *Fulbrightiella, Machochaete* | *Calothrix* PCC-6303 | 0 | 0 | 0 | 0 | 0 | 0 | 0 | 240 | 16 | 0 | 0 | 256 | 0,16 |
| *Microchaete* | *Desmonostoc* PCC-7422 | 0 | 0 | 0 | 0 | 0 | 0 | 0 | 0 | 72 | 0 | 0 | 72 | 0,05 |
| *Nodularia* | *Nodularia* PCC-9350 | 0 | 0 | 0 | 0 | 0 | 48 | 0 | 532 | 0 | 0 | 0 | 580 | 0,37 |
| *Mojavia, Nostoc,* Unknown genus 6 | *Nostoc* PCC-73102 | 0 | 0 | 16436 | 15884 | 0 | 2449 | 0 | 3738 | 13924 | 26284 | 12066 | 90781 | 57,42 |
| *Purpureonostoc, Pseudoaliinostoc* | *Nostoc* PCC-7524 | 0 | 0 | 87 | 0 | 0 | 0 | 0 | 443 | 168 | 0 | 0 | 698 | 0,44 |
| *Cyanocohniella, Anabaena, Trichormus, Dulcicalothrix, Cylindrospermum* | Nostocaceae | 0 | 0 | 0 | 0 | 0 | 24 | 0 | 60 | 0 | 0 | 0 | 84 | 0,05 |
| *Rivularia* | *Rivularia* PCC-7116 | 0 | 6633 | 21 | 41 | 13071 | 0 | 0 | 0 | 0 | 0 | 0 | 19766 | 12,50 |
| Unknown genus 2 | Oscillatoriaceae | 0 | 1868 | 0 | 0 | 0 | 0 | 0 | 0 | 0 | 0 | 0 | 1868 | 1,18 |
| *Jaaginema* | Paraspirulinaceae | 353 | 0 | 0 | 0 | 0 | 0 | 0 | 0 | 0 | 0 | 0 | 353 | 0,22 |
| Unknown genus 1, *Limnoraphis, Microcoleus, Salileptolyngbya* | Phormidiaceae | 0 | 0 | 0 | 0 | 0 | 0 | 17452 | 0 | 0 | 0 | 0 | 17452 | 11,04 |
| *Phormidesmis, U*nknown genus 9, Unknown genus 16 | Leptolyngbyaceae | 0 | 0 | 0 | 0 | 0 | 0 | 226 | 0 | 0 | 0 | 0 | 226 | 0,14 |
| *Chamaesiphon* | *Calothrix* KVSF5 | 0 | 0 | 0 | 0 | 0 | 0 | 0 | 0 | 0 | 11 | 0 | 11 | 0,01 |
| *Thermoleptolyngbya* | *Geitlerinema* PCC-8501 | 0 | 0 | 10 | 0 | 0 | 0 | 0 | 0 | 0 | 0 | 0 | 10 | 0,01 |
| *Timaviella* | *Phormidium* CYN64 | 0 | 0 | 0 | 0 | 0 | 121 | 263 | 0 | 0 | 0 | 0 | 384 | 0,24 |
| *Toxifilum* | Unknown Family | 0 | 148 | 0 | 0 | 213 | 0 | 0 | 18 | 0 | 0 | 0 | 379 | 0,24 |
| *Nodosilinea, Haloleptolyngbya* | *Nodosilinea* PCC-7104 | 0 | 0 | 114 | 0 | 298 | 0 | 0 | 33 | 16 | 0 | 54 | 515 | 0,33 |
| Unknown genus 5 | *Synechococcus* PCC-7502 | 0 | 0 | 27 | 0 | 0 | 0 | 0 | 0 | 0 | 0 | 0 | 27 | 0,02 |
| *Altericista* | SepB-3 | 0 | 0 | 148 | 0 | 0 | 0 | 0 | 0 | 0 | 0 | 0 | 148 | 0,09 |
| *Cyanobium* | *Cyanobium* PCC-6307 | 0 | 0 | 569 | 0 | 0 | 33 | 0 | 17 | 36 | 0 | 0 | 655 | 0,41 |
| *Parasynechococcus* | *Synechococcus* sp. | 0 | 0 | 587 | 0 | 0 | 0 | 0 | 0 | 0 | 0 | 0 | 587 | 0,37 |
| Unknown genus 3 | *Schizothrix* LEGE | 0 | 1115 | 0 | 51 | 319 | 0 | 0 | 0 | 0 | 0 | 0 | 1485 | 0,94 |
| Unknown genus 10 | Sericytochromatia | 0 | 59 | 11 | 0 | 0 | 0 | 0 | 31 | 0 | 0 | 0 | 101 | 0,06 |
| Total reads of Cyanophyceae |  | 21719 | 9843 | 18230 | 15976 | 13911 | 2687 | 17941 | 5151 | 14232 | 26295 | 12120 | 158105 |  |
| Relative abundance in the Cyanophyceae community |  | 13,7 | 6,2 | 11,5 | 10,1 | 8,7 | 1,6 | 11,3 | 3,2 | 9,0 | 16,6 | 7,62 |  |  |
| Total amplicon reads (Bacteria community) |  | 59629 | 72743 | 57566 | 57435 | 76498 | 90816 | 79104 | 73051 | 85853 | 69603 | 64537 | 786835 |  |
