# Supplementary material for: Cyanobacterial mats and their associated microbiomes in saline and freshwater lakes from the Bolivian Altiplano
Source: Front Microbiol. 2025 Jul 23;16:1650455. doi: 10.3389/fmicb.2025.1650455 (PMC12325342; doi:10.3389/fmicb.2025.1650455)
Supplement: Supplementary file 2 [file Table_2.docx]

Table S2. 16S rRNA gene phylogenetic analysis and identity (p-distance) results, supporting genus-level identification of cyanobacterial sequences obtained in this study.

| Sequence numbers (correspond to the sequence labels in Fig. 4) | Phylogenetic closest related reference strain | 16S identity with closest related reference strain(%)* | Final genus identification | Order |
| --- | --- | --- | --- | --- |
| 1 | *Mojavia pulchra* JT2-VF2 (AY577534) | 97.52 | *Mojavia* | Nostocales |
| 2 | *Rivularia halophila* PUNANP3PCI185B (KY296608) | 99.75 | *Rivularia* | Nostocales |
| 3 | *Coleofasciculus chthonoplastes* GNL1 (EF654033) | 100 | *Coleofasciculus* | Coleofasciculales |
| 4 | *Kamptonema animale* CCALA771 (KP221932) | 94.83 | Unknown genus 1 (with 70) | Oscillatoriales |
| 5 | *Nostoc commune* EV1-KK1 (AY577536) | 100 | *Nostoc* | Nostocales |
| 6 | *Nostoc commune* EV1-KK1 (AY577536) | 98.76 | *Nostoc* | Nostocales |
| 7 | *Nostoc commune* EV1-KK1 (AY577536) | 99.50 | *Nostoc* | Nostocales |
| 8 | *Nostoc commune* EV1-KK1 (AY577536) | 99.50 | *Nostoc* | Nostocales |
| 9 | *Nostoc commune* EV1-KK1 (AY577536) | 98.76 | *Nostoc* | Nostocales |
| 10 | Coleofasciculus chthonoplastes GNL1 (EF654033) | 99.75 | *Coleofasciculus* | Coleofasciculales |
| 11 | *Nostoc commune* EV1-KK1 (AY577536) | 99.75 | *Nostoc* | Nostocales |
| 12 | *Neolyngbya maris-brasilis* ALCB114381 (KY824055) | 89.71 | Unknown genus 2 (with 43) | Oscillatoriales |
| 13 | *Cyanobium gracile* PCC6307 (CP003495) | 88.86 | Unknown genus 3 (with 108 and 198) | Chroococcales |
| 14 | *Mojavia pulchra* JT2-VF2 (AY577534) | 97.28 | *Mojavia* | Nostocales |
| 15 | *Nostoc commune* EV1-KK1 (AY577536) | 99.50 | *Nostoc* | Nostocales |
| 16 | *Parasynechococcus* WH8109 (AY172836) | 96.29 | *Parasynechococcus* | Chroococcales |
| 17 | Nostocales cyanobacterium CHAB5880 (MN381942) | 96.82 | *Purpureonostoc* | Nostocales |
| 18 | *Nodularia* PCC9350 (AY038034) | 99.75 | *Nodularia* | Nostocales |
| 19 | *Toxifilum mysidocida* MYSIDO1 (KX375342) | 99.50 | *Toxifilum* | Oculatellales |
| 20 | *Jaaginema* PsrJGgm14 (KM438189) | 95.31 | *Jaaginema* | Leptolyngbyales |
| 21 | *Nodosilinea nodulosa* PCC7104 (AB039012) | 98.27 | *Nodosilinea* | Nodosilineales |
| 22 | *Fulbrightiella* XP11C (AM230698) | 100 | *Fulbrightiella* | Nostocales |
| 23 | *Nostoc commune* EV1-KK1 (AY577536) | 99.75 | *Nostoc* | Nostocales |
| 24 | *Cyanobium gracile* PCC6307 (CP003495) | 100 | *Cyanobium* | Chroococcales |
| 25 | *Cyanobium gracile* PCC6307 (CP003495) | 98.27 | *Cyanobium* | Chroococcales |
| 27 | *Timaviella karstica* GR13 (LT634150) | 96.79 | *Timaviella* | Oculatellales |
| 28 | *Cyanobium gracile* PCC6307 (CP003495) | 98.02 | *Cyanobium* | Chroococcales |
| 29 | *Altericista variichlora* CALU1173 (MT463167) | 94.55 | Unknown genus 15 | Synechococcales |
| 30 | *Phormidesmis arctica* HOR116 (KU219729) | 97.91 | *Phormidesmis* | Leptolyngbyales |
| 31 | *Timaviella karstica* GR13 (LT634150) | 98.52 | *Timaviella* | Oculatellales |
| 33 | *Mojavia pulchra* JT2-VF2 (AY577534) | 97.28 | *Mojavia* | Nostocales |
| 35 | Nostocales cyanobacterium CHAB5880 (MN381942) | 98.778 | *Purpureonostoc* | Nostocales |
| 38 | *Pseudoaliinostoc sejongens* ACKU594 (MT000689) | 98.51 | *Pseudoaliinostoc* | Nostocales |
| 41 | *Microchaete tenera* ACOI1451 (HE797732) | 98.44 | *Microchaete* | Nostocales |
| 42 | *Timaviella karstica* GR13 (LT634150) | 96.54 | *Timaviella* | Oculatellales |
| 43 | *Neolyngbya maris-brasilis* ALCB114381 (KY824055) | 92.1 | Unknown genus 2 (with 12) | Oscillatoriales |
| 44 | *Phormidesmis arctica* HOR116 (KU219729) | 98.17 | *Phormidesmis* | Leptolyngbyales |
| 45 | *Coelomoron pusillum* AICB1012 (KJ746507) | 92.59 | Unknown genus 10 | Chroococcales |
| 50 | *Nodosilinea nodulosa* PCC7104 (AB039012) | 98.27 | *Nodosilinea* | Nodosilineales |
| 51 | *Nodosilinea nodulosa* PCC7104 (AB039012) | 98.27 | *Nodosilinea* | Nodosilineales |
| 54 | *Nodosilinea nodulosa* PCC7104 (AB039012) | 98.76 | *Nodosilinea* | Nodosilineales |
| 55 | *Jaaginema* PsrJGgm14 (KM438189) | 95.31 | *Jaaginema* | Leptolyngbyales |
| 56 | *Cyanocohniella calida* CCALA1049 (KJ737428) | 99.26 | *Cyanocohniella* | Nostocales |
| 57 | *Potamosiphon austaliensis* FHC091403 (MH047862) | 98.02 | *Potamosiphon* | Oscillatoriales |
| 58 | *Anagnostidinema pseudacutissimum* CCALA 150 (KT315940) | 98.51 | *Anagnostidinema* | Coleofasciculales |
| 60 | *Nodularia* PCC9350 (AY038034) | 99.51 | *Nodularia* | Nostocales |
| 63 | *Nostoc commune* EV1-KK1 (AY577536) | 99.75 | *Nostoc* | Nostocales |
| 65 | *Coleofasciculus chthonoplastes* GNL1 (EF654033) | 99.75 | *Coleofasciculus* | Coleofasciculales |
| 67 | *Romeriopsis marina* LEGE06013 (MN537584) | 88.59 | Unknown genus 9 | Leptolyngbyales |
| 68 | *Cyanobium gracile* PCC6307 (CP003495) | 99.26 | *Cyanobium* | Chroococcales |
| 70 | *Kamptonema animale* CCALA771 (KP221932) | 94.58 | Unknown genus 1 (with 4) | Oscillatoriales |
| 73 | *Coelomoron pusillum* AICB1012 (KJ746507) | 93.58 | Unknown genus 10 | Chroococcales |
| 74 | *Mojavia pulchra* JT2-VF2 (AY577534) | 97.28 | *Mojavia* | Nostocales |
| 76 | *Nodularia* PCC9350 (AY038034) | 99.26 | *Nodularia* | Nostocales |
| 78 | *Microcystis aeruginosa* NIES1355 (LC557445) | 98.02 | *Microcystis* | Chroococcales |
| 82 | *Nostoc commune* EV1-KK1 (AY577536) | 98.51 | *Nostoc* | Nostocales |
| 84 | *Nostoc commune* EV1-KK1 (AY577536) | 99.49 | *Nostoc* | Nostocales |
| 88 | *Coleofasciculus chthonoplastes* GNL1 (EF654033) | 99.75 | *Coleofasciculus* | Coleofasciculales |
| 91 | *Nostoc commune* EV1-KK1 (AY577536) | 99.75 | *Nostoc* | Nostocales |
| 92 | *Nostoc commune* EV1-KK1 (AY577536) | 99.26 | *Nostoc* | Nostocales |
| 93 | *Nostoc commune* EV1-KK1 (AY577536) | 99.26 | *Nostoc* | Nostocales |
| 95 | *Cyanobium gracile* PCC6307 (CP003495) | 97.28 | *Cyanobium* | Chroococcales |
| 96 | *Hillbrichtia pamiria* TAUMACCylx15 (NR176595) | 91.60 | Unknown genus 4 | Coleofasciculales |
| 98 | *Tumidithrix elongata* BACA0141 (MT176747) | 93.78 | Unknown genus 5 (with 199) | Pseudanabaenales |
| 99 | *Nodosilinea nodulosa* PCC7104 (AB039012) | 98.02 | *Nodosilinea* | Nodosilineales |
| 104 | *Synechocystis* PCC6714 (AB041937) | 98.02 | *Synechocystis* | Chroococcales |
| 107 | *Rivularia halophila* PUNANP3PCI185B (KY296608) | 88.12 | Unknown genus 6 (with 261, 122, 241, 221, 301, 181) | Nostocales |
| 108 | *Cyanobium gracile* PCC6307 (CP003495) | 90.10 | Unknown genus 3 (with 13 and 198) | Chroococcales |
| 116 | *Nodosilinea nodulosa* PCC7104 (AB039012) | 99.50 | *Nodosilinea* | Nodosilineales |
| 117 | *Timaviella karstica* GR13 (LT634150) | 96.30 | *Timaviella* | Oculatellales |
| 118 | *Limnococcus limneticus* Svet06 (GQ375048) | 93.84 | Unknown genus 7 (with 239) | Chroococcales |
| 122 | *Rivularia halophila* PUNANP3PCI185B (KY296608) | 92.18 | Unknown genus 6 (with 261, 107, 241, 221, 301, 181) | Nostocales |
| 124 | *Nostoc commune* EV1-KK1 (AY577536) | 99.01 | *Nostoc* | Nostocales |
| 125 | *Anabaena oscillarioides* BECID32 (AJ630427) | 99.51 | *Anabaena* | Nostocales |
| 126 | *Limnoraphis robusta* CCALA966 (NR118325) | 100 | *Limnoraphis* | Oscillatoriales |
| 127 | *Nodosilinea nodulosa* PCC7104 (AB039012) | 97.52 | *Nodosilinea* | Nodosilineales |
| 133 | *Mojavia pulchra* JT2-VF2 (AY577534) | 97.52 | *Mojavia* | Nostocales |
| 145 | *Anabaenopsis elenkinii* AB2006/20 | 90.42 | Unknown genus 8 | Nostocales |
| 146 | *Nostoc commune* EV1-KK1 (AY577536) | 95.05 | *Nostoc* | Nostocales |
| 147 | *Haloleptolyngbya alcalis* KR2005106 (JN712770) | 95.06 | *Haloleptolyngbya* | Synechococcales |
| 148 | *Geitlerinema splendidum* CCALA150 (KT315940) | 99.50 | *Anagnostidinema* | Coleofasciculales |
| 157 | *Fulbrightiella* XP11C (AM230698) | 100 | *Fulbrightiella* | Nostocales |
| 158 | *Limnococcus limneticus* Svet06 (GQ375048) | 96.07 | *Limnococcus* | Chroococcales |
| 160 | *Chamaesiphon polonicus* SAG3287 (KM019983) | 96.56 | *Chamaesiphon* | Gomontiellales |
| 161 | *Nostoc commune* EV1-KK1 (AY577536) | 99.26 | *Nostoc* | Nostocales |
| 162 | *Rivularia halophila* PUNANP3PCI185B (KY296608) | 97.97 | *Rivularia* | Nostocales |
| 167 | *Nodosilinea nodulosa* PCC7104 (AB039012) | 97.28 | *Nodosilinea* | Nodosilineales |
| 168 | *Leptolyngbya laminose* ETS08 (FM210757) | 98.76 | *Thermoleptolyngbya* | Oculatellales |
| 169 | Uncultured bacterium FE203 (KC294737) | 97.03 | *Crocosphaera* | Chroococcales |
| 178 | *Trichormus variabilis* HINDAK2001/4 (AJ630456) | 99.51 | *Trichormus* | Nostocales |
| 179 | *Ancylothrix rivularis* 8PC (KT819197) | 95.31 | *Ancylothrix* | Oscillatoriales |
| 181 | *Rivularia halophila* PUNANP3PCI185B (KY296608) | 90.52 | Unknown genus 6 (with 261, 122, 107, 241, 301, 221) | Nostocales |
| 195 | *Dulcicalothrix necridiiformans* V1316S (KY863521) | 94.79 | Unknown genus 12 (with 209) | Nostocales |
| 196 | *Odorella benthonica* CalAq792 (MH702368) | 100 | *Odorella* | Chroococcales |
| 197 | *Limnococcus limneticus* Svet06 (GQ375048) | 96.32 | *Limnococcus* | Chroococcales |
| 198 | *Cyanobium gracile* PCC6307 (CP003495) | 89.11 | Unknown genus 3 (with 13 and 108) | Chroococcales |
| 199 | *Tumidithrix elongata* BACA0141 (MT176747) | 93.28 | Unknown genus 5 | Pseudanabaenales |
| 200 | *Nodularia* PCC9350 (AY038034) | 99.51 | *Nodularia* | Nostocales |
| 208 | *Synechocystis* PCC6714 (AB041937) | 97.78 | *Synechocystis* | Chroococcales |
| 209 | *Dulcicalothrix necridiiformans* V1316S (KY863521) | 94.54 | Unknown genus 12 (with 295) | Nostocales |
| 210 | *Macrochaete psychrophila* CCALA1092 (KR350578) | 99.50 | *Macrochaete* | Nostocales |
| 218 | *Microcoleus vaginatus* SAG2211 (EF654074) | 98.56 | *Microcoleus* | Oscillatoriales |
| 221 | *Rivularia halophila* PUNANP3PCI185B (KY296608) | 87.31 | Unknown genus 6 (with 261, 122, 107, 241, 301, 181) | Nostocales |
| 222 | *Scytolyngbya timoleontis* XS01 (KP688591) | 93.10 | Unknown genus 16 | Leptolyngbyales |
| 223 | *Chamaesiphon polonicus* SAG3287 (KM019983) | 97.04 | *Chamaesiphon* | Gomontiellales |
| 224 | *Salileptolyngbya diazotrophicum* SCSIO43686 (MF614799) | 97.28 | *Salileptolyngbya* | Nodosilineales |
| 234 | Uncultured bacterium FE203 (KC294737) | 96.53 | *Crocosphaera* | Chroococcales |
| 235 | *Arthrospira platensis* SAG8579 (KM019968) | 99.26 | *Limnospira* | Oscillatoriales |
| 236 | *Coleofasciculus chthonoplastes* GNL1 (EF654033) | 86.7 | *Coleofasciculus* | Coleofasciculales |
| 237 | *Phormidesmis arctica* HOR116 (KU219729) | 89.09 | Unknown genus 11 | Leptolyngbyales |
| 238 | *Aphanothece sacrum* (AB116658) | 99.51 | *Aphanothece* | Chroococcales |
| 239 | *Limnococcus limneticus* Svet06 (GQ375048) | 93.6 | Unknown genus 7 (with 118) | Chroococcales |
| 240 | *Speleotes anchialus* TAUMAC1118 (OL310683) | 99.01 | *Speleotes* | Chroococcales |
| 241 | *Rivularia halophila* PUNANP3PCI185B (KY296608) | 85.68 | Unknown genus 6 (with 261, 122, 107, 221, 301, 181) | Nostocales |
| 244 | *Phormidesmis arctica* MUM118 (KU219738) | 93.49 | Unknown genus 13 | Leptolyngbyales |
| 251 | *Pycnacronema brasiliensis* 45PC (MF581661) | 96.54 | *Pycnacronema* | Coleofasciculales |
| 252 | *Cylindrospermum alatosporum* CCALA994 (KF052609) | 99.01 | *Cylindrospermum* | Nostocales |
| 260 | *Coelomoron pusillum* AICB1012 (KJ746507) | 93.33 | Unknown genus 14 | Chroococcales |
| 261 | *Rivularia halophila* PUNANP3PCI185B (KY296608) | 87.90 | Unknown genus 6 (with 122, 107, 241, 221, 301, 181) | Nostocales |
| 262 | *Geminocystis herdmanii* PCC6308 (AB039001) | 95.78 | *Geminocystis* | Chroococcales |
| 263 | *Heteroleibleinia purpurascens* GR2 (LT634148) | 96.53 | *Heteroleibleinia* | Leptolyngbyales |
| 270 | *Nostoc commune* EV1-KK1 (AY577536) | 99.5 | *Nostoc* | Nostocales |
| 277 | *Coleofasciculus chthonoplastes* GNL1 (EF654033) | 91.29 | *Coleofasciculus* | Coleofasciculales |
| 290 | *Pycnacronema brasiliensis* 45PC (MF581661) | 99.51 | *Pycnacronema* | Coleofasciculales |
| 295 | *Stenomitos rutilans* HA7619LM2 (KF417430) | 98.51 | *Stenomitos* | Leptolyngbyales |
| 296 | *Pycnacronema brasiliensis* 45PC (MF581661) | 99.26 | *Pycnacronema* | Coleofasciculales |
| 298 | *Nodosilinea ramsarensis* KHSS26 (MF348321) | 94.10 | *Nodosilinea* | Nodosilineales |
| 299 | *Altericista variichlora* CALU1173 (MT463167) | 83.82 | Unknown genus 15 | Synechococcales |
| 301 | *Rivularia halophila* PUNANP3PCI185B (KY296608) | 93.15 | Unknown genus 6 (with 122, 107, 241, 221, 261, 181) | Nostocales |
| 302 | *Anagnostidinema pseudacutissimum* CCALA 150 (KT315940) | 98.76 | *Anagnostidinema* | Coleofasciculales |
| 303 | *Coleofasciculus chthonoplastes* GNL1 (EF654033) | 97.05 | *Coleofasciculus* | Coleofasciculales |
| 304 | *Coleofasciculus chthonoplastes* GNL1 (EF654033) | 98.58 | *Coleofasciculus* | Coleofasciculales |
| 305 | *Rivularia halophila* PUNANP3PCI185B (KY296608) | 99.64 | *Rivularia* | Nostocales |
| 306 | *Rivularia halophila* PUNANP3PCI185B (KY296608) | 95.10 | *Rivularia* | Nostocales |
| 308 | *Rivularia halophila* PUNANP3PCI185B (KY296608) | 99.02 | *Rivularia* | Nostocales |

*similarity below 95% is indicative of distinct genera
