# Supplementary figures and images for: Cyanobacterial mats and their associated microbiomes in saline and freshwater lakes from the Bolivian Altiplano
Source: Front Microbiol. 2025 Jul 23;16:1650455. doi: 10.3389/fmicb.2025.1650455 (PMC12325342; doi:10.3389/fmicb.2025.1650455)

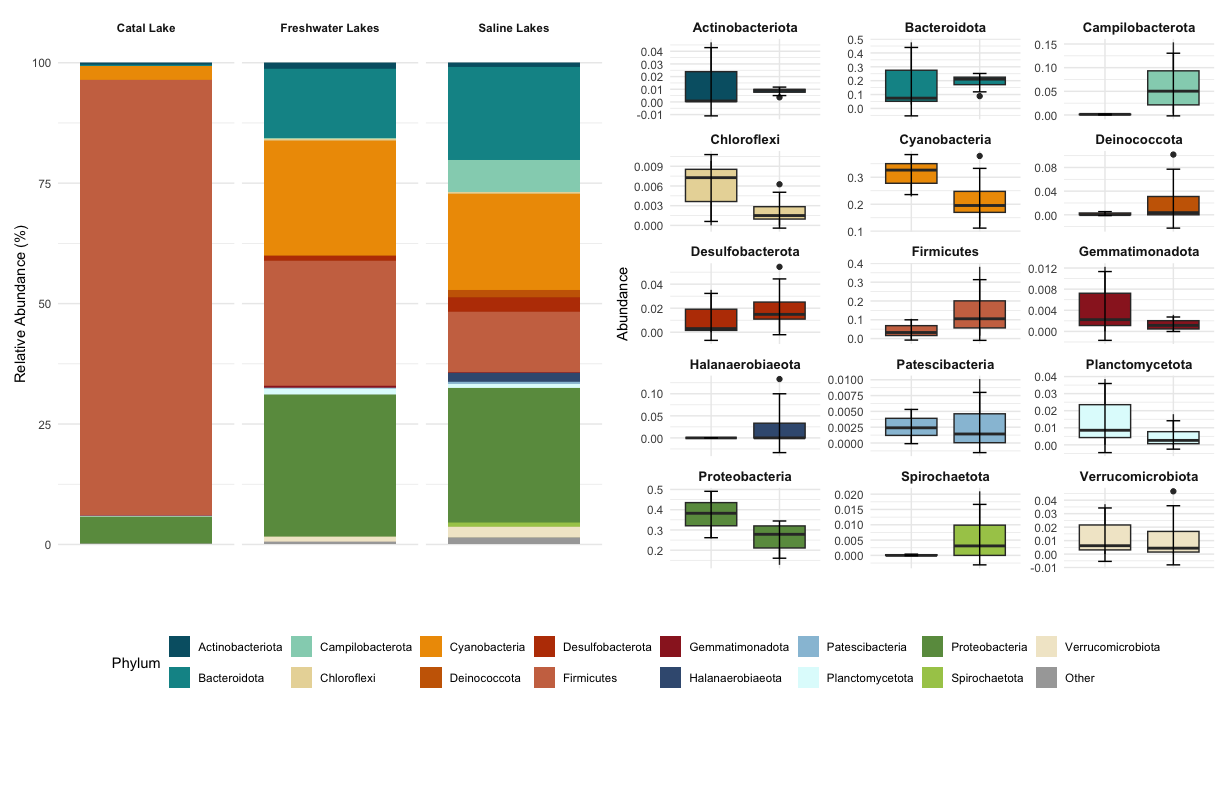

Supplement: Supplementary file 4 [file Image_1.tiff]

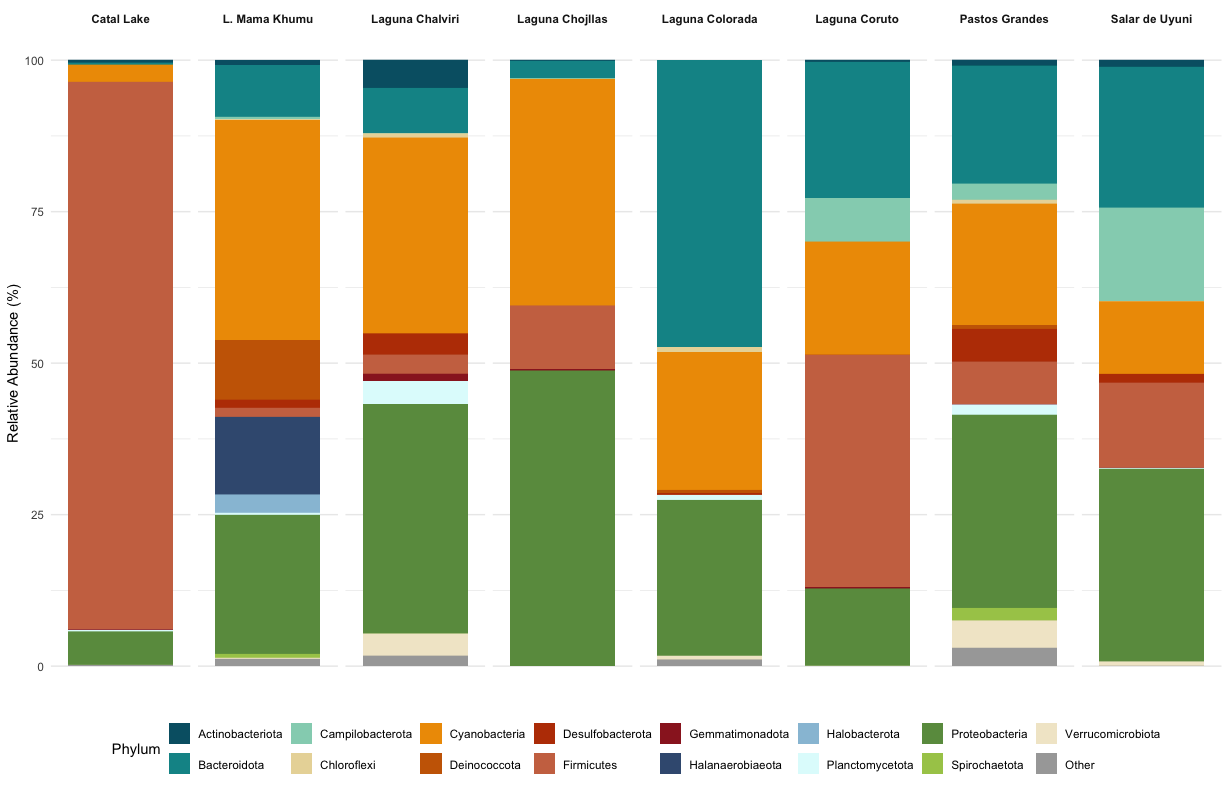

Supplement: Supplementary file 5 [file Image_2.tiff]

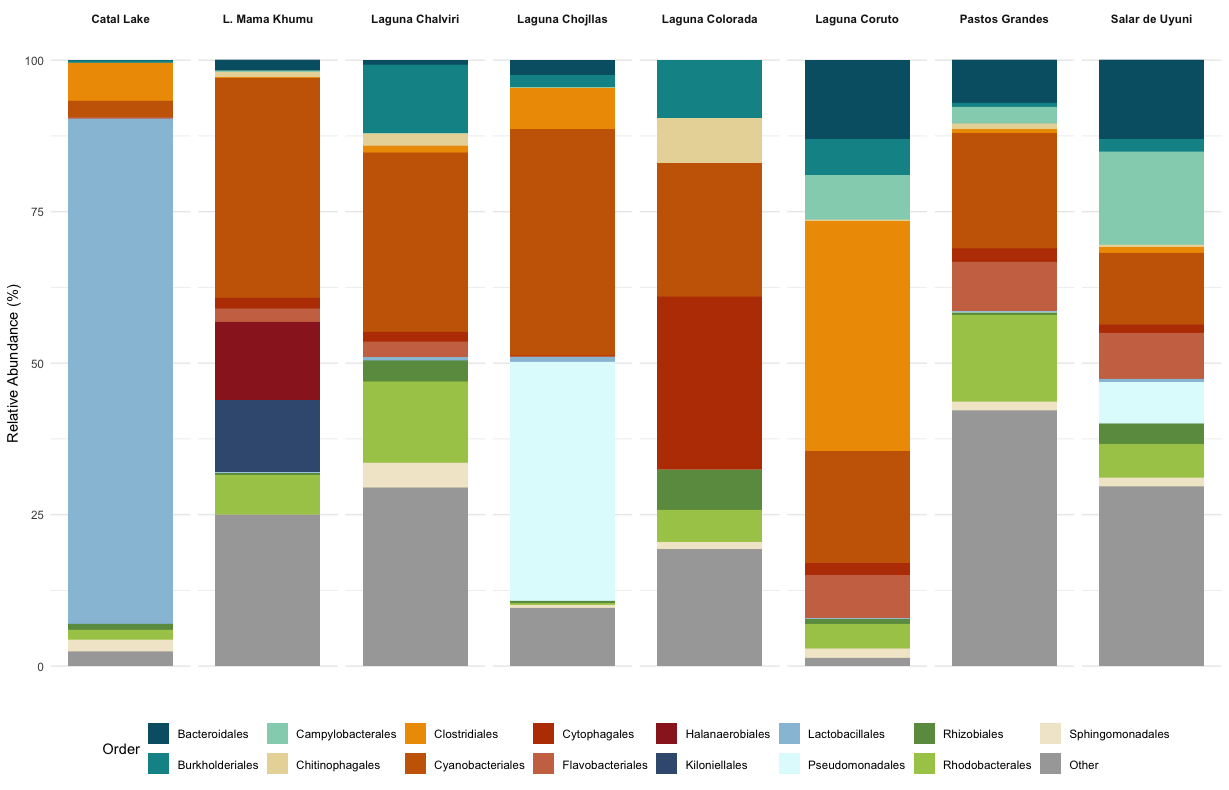

Supplement: Supplementary file 6 [file Image_3.tiff]

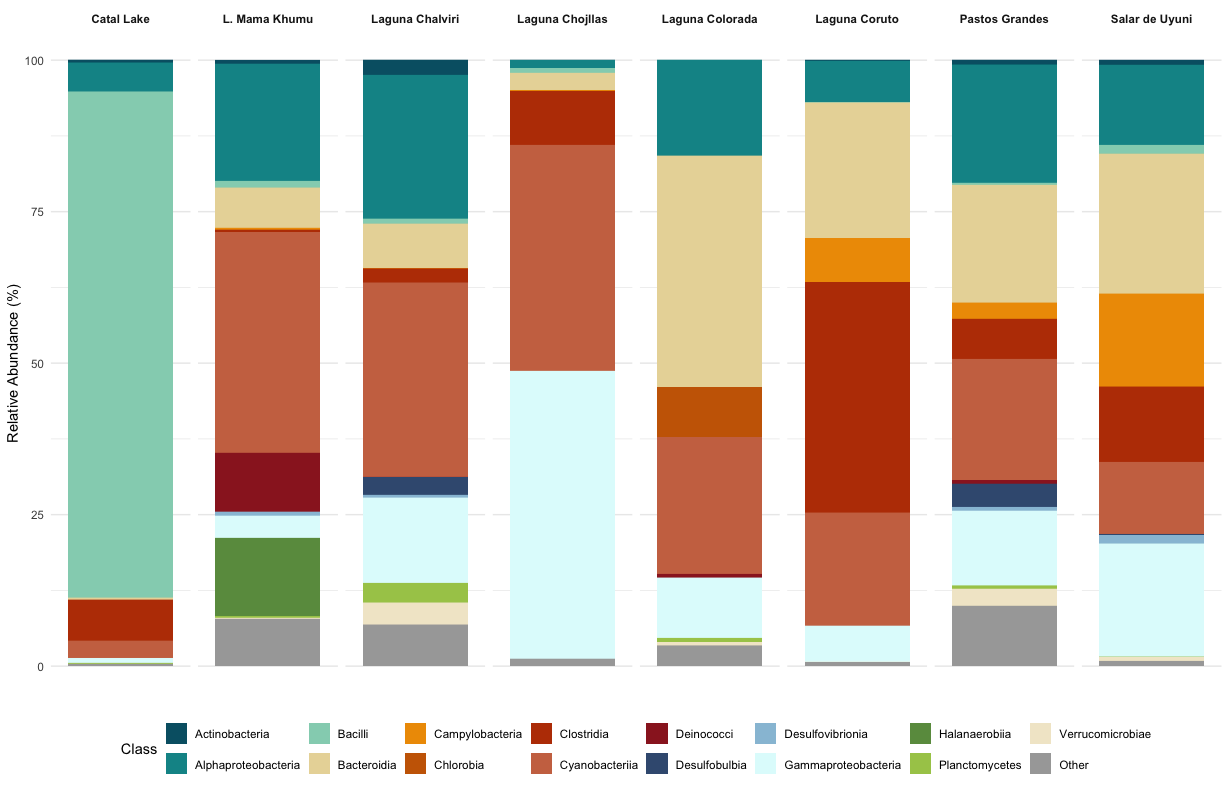

Supplement: Supplementary file 7 [file Image_4.tiff]

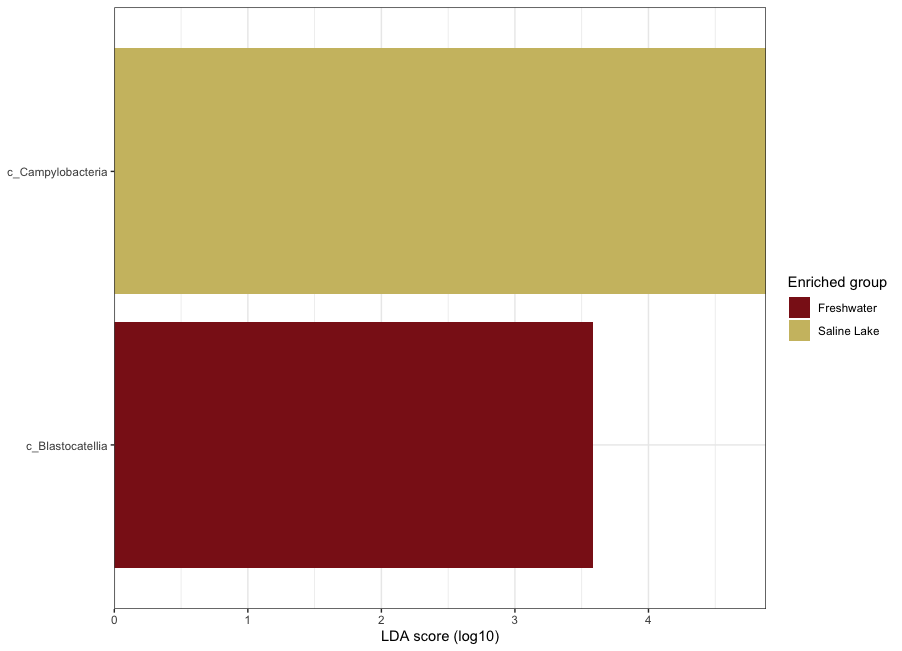

Supplement: Supplementary file 8 [file Image_5.tiff]
